# Visualizing Dynamic Changes at the Maternal-Fetal Interface Throughout Human Pregnancy by Mass Cytometry

**DOI:** 10.3389/fimmu.2020.571300

**Published:** 2020-10-26

**Authors:** Anita van der Zwan, Vincent van Unen, Guillaume Beyrend, Sandra Laban, Carin van der Keur, Hanneke J. M. Kapsenberg, Thomas Höllt, Susana M. Chuva de Sousa Lopes, Marie-Louise P. van der Hoorn, Frits Koning, Frans H. J. Claas, Michael Eikmans, Sebastiaan Heidt

**Affiliations:** ^1^Department of Immunology, Leiden University Medical Center, Leiden, Netherlands; ^2^Institute for Immunity, Transplantation and Infection, Stanford University School of Medicine, Stanford, CA, United States; ^3^Leiden Computational Biology Center, Leiden University Medical Center, Leiden, Netherlands; ^4^Computer Graphics and Visualization Group, Delft University of Technology, Delft, Netherlands; ^5^Department of Anatomy and Embryology, Leiden University Medical Center, Leiden, Netherlands; ^6^Department of Obstetrics and Gynaecology, Leiden University Medical Center, Leiden, Netherlands

**Keywords:** immune profiling, human atlas, pregnancy, placenta, decidua, peripheral blood

## Abstract

During healthy pregnancy, a balanced microenvironment at the maternal-fetal interface with coordinated interaction between various immune cells is necessary to maintain immunological tolerance. While specific decidual immune cell subsets have been investigated, a system-wide unbiased approach is lacking. Here, mass cytometry was applied for data-driven, in-depth immune profiling of the total leukocyte population isolated from first, second, and third trimester decidua, as well as maternal peripheral blood at time of delivery. The maternal-fetal interface showed a unique composition of immune cells, different from peripheral blood, with significant differences between early and term pregnancy samples. Profiling revealed substantial heterogeneity in the decidual lymphoid and myeloid cell lineages that shape gestational-specific immune networks and putative differentiation trajectories over time during gestation. Uncovering the overall complexity at the maternal-fetal interface throughout pregnancy resulted in a human atlas that may serve as a foundation upon which comprehension of the immune microenvironment and alterations thereof in pregnancy complications can be built.

## Introduction

Preserving immunological tolerance toward the semi-allogeneic fetus during pregnancy while providing protection against environmental pathogens relies on intricately regulated local and systemic immune adaptations. Direct contact between the mother and the fetus exists at the decidua basalis, located at the implantation site, and at the decidua parietalis that is part of the membranes which line the uterine cavity and surround the fetus. Fetal extravillous trophoblasts (EVT) migrate into the maternal decidua early during pregnancy (1), and express HLA-C, -G, -E, and -F but lack expression of the classical HLA-A and -B antigens, rendering them in part invisible to natural killer (NK) cells and the large majority of maternal allogeneic CD8+ T cells (2–4). In concert, alterations in both the maternal innate and adaptive immune compartment occur, where NK and innate lymphoid cells (ILC) prevail in early pregnancy, while T cell proportions increase over the course of gestation (5, 6). Antigen-presenting cell (APC) numbers remain relatively constant throughout pregnancy while B cells have been described as a sparse population (5–8).

The fetus can be immunologically recognized as maternal NK cells may bind to fetal HLA-C and HLA-G, and fetal-specific CD8+ and CD4+ T cells have been observed in maternal peripheral blood and decidua (9–12). As such, aberrant regulation of the maternal immune system has been suggested to play a role in pregnancy complications, such as pre-eclampsia (13, 14), recurrent miscarriages (15, 16), preterm birth (17–19), and fetal growth restrictions (20).

A better understanding of the immune system at the maternal-fetal interface during a healthy pregnancy may drive the systematic investigation of major pregnancy complications. Most work in the field of reproductive immunology has focused on individual subsets of decidual immune cells while a comprehensive, system-wide approach that visualizes all decidual immune cell lineages at different time points during pregnancy is lacking. High-dimensional single-cell technologies such as mass cytometry (21) allow an in-depth and unbiased data-driven analysis of the composition of the immune system at the maternal-fetal interface. In the current study, we applied two mass cytometry antibody panels, one to detect heterogeneity within all major immune cell lineages while the other with a focus on T cell-specific markers, to determine the composition of the maternal immune compartment in first, second, and third (term) trimester decidual samples as well as maternal PBMC (mPBMC) at the time of delivery. Our results provide an immune atlas of the maternal-fetal interface in healthy pregnancy, which may serve as a foundation for improved understanding of pregnancy complications.

## Materials and Methods

### Human Decidual and Blood Samples

De-identified 1st and 2nd trimester human decidual material (1st trimester, gestational age of 6–13 weeks, *n* = 12; 2nd trimester, 14–18 weeks, *n* = 6) was obtained from women undergoing elective pregnancy termination. The gestational age was determined by ultrasonography and the tissue obtained by vacuum aspiration. Paired 3rd trimester (term) decidua basalis, decidua parietalis, and heparinized mPBMC were obtained from healthy women after uncomplicated pregnancy (gestational age >38 weeks, *n* = 9) delivered by elective cesarean section or uncomplicated spontaneous vaginal delivery at Leiden University Medical Center (LUMC). Non-pregnant PBMC control samples were obtained from healthy females (*n* = 4). The clinical characteristics of the subjects are shown in Table 1. All samples were obtained after informed consent and the study was carried out in accordance with the guidelines issued by the Medical Ethics Committee of the LUMC (protocols P08.087 and P11.196), and in accordance with the Declaration of Helsinki.

**TABLE 1:** Patient characteristics^1^.

	**1st trimester**	**2nd trimester**	**3rd trimester**	**NP PBMC**
**Demographics**				
Maternal age (years; mean ± SD)	Unknown	Unknown	32.8 ± 4.3	30.5 ± 3.1
Body mass index (BMI; mean ± SD)	Unknown	Unknown	25.4 ± 3.9	22.1 ± 0.4
Gravity (median, IQR) ^2^	Unknown	Unknown	2 (1, 2)	0
Parity (% nulliparous)	Unknown	Unknown	46	100
**Pregnancy parameters**				
Gestational age (weeks; mean ± SD)	9.2 ± 2.1	15.5 ± 1.2	39.1 ± 0.8	NA
Placenta weight (kg; mean ± SD)	NA	NA	564.5 ± 87.3	NA
Mode of delivery	Elective abortion	Elective abortion	Spontaneous + C-section	NA
Sex of child (%)	M 53.8%/ F 46.2%	M 9.1%/ F 90.9%	M 53.8%/ F 46.2%	NA
**Experiment inclusions**				
General CyTOF panel	*n* = 12	*n* = 6	*n* = 9	*n* = 4
T cell CyTOF panel	*n* = 11	*n* = 5	*n* = 8	*n* = 4
FACS panel	n = 3	n = 4	n = 4	NA

*^1^All pregnancies were considered healthy as determined by demographics, pregnancy parameters, attending gynecologists/research nurses, and the absence of membrane discoloration and infarctions in the placenta. ^2^ IQR, interquartile range.*

### Isolation of Lymphocytes From Decidual and PBMC Samples

Decidual leukocytes were isolated as previously described, with some adjustments (22). For isolation of 1st and 2nd trimester decidual leukocytes, villous and decidual tissues from elective pregnancy terminations were macroscopically identified and separated. Decidua basalis and parietalis from term pregnancy were macroscopically dissected by scraping the basalis membrane from the placenta and by removing the amnion and delicately scraping the decidua parietalis from the chorion. Decidual tissues were washed with PBS, minced, and resuspended in Accutase cell detachment solution (prewarmed to 37°C; Gibco Life technologies). Subsequently, tissues were transferred to a C tube, homogenized on a gentleMACS dissociator (Miltenyi Biotec Ltd.) and incubated for 60 min in a water bath (37°C, gently shaking), at 30 min spinning the C tubes once more. After digestion, released cell suspensions were filtered through 250 and 70 μm sieves (Sigma-Aldrich; Miltenyi Biotec Ltd.) and washed with RPMI 1640 (Life technologies). Next, the cell suspensions were dissolved in 20 ml of 1.023 g/ml Percoll (GE Healthcare) and layered on a Percoll gradient (10 ml 1.080 g/ml; 15 ml 1.053 g/ml) for density gradient centrifugation (25 min, 2000 rpm). Leukocytes were isolated from the 1.080–1.053 g/ml and the 1.053–1.023 g/ml interface, washed twice with RPMI, and left overnight at 4°C. Peripheral blood leukocytes were isolated from freshly drawn heparin anticoagulated blood using Ficoll (GE Healthcare) density gradient centrifugation (20 min, 2000 rpm) and left overnight at 4°C. The next day, cell suspensions were incubated with Benzonase Nuclease (Sigma-Aldrich; 20U/mL) for 5 min, washed, counted, and stained with antibodies for either mass cytometry or flow cytometry. To account for cell processing variation, the effects of enzymatic digestion and gentleMACS dissociation on cell surface protein markers in peripheral blood and decidual cell suspensions has extensively been validated in our laboratory and by others (23).

### Mass Cytometry Antibody Staining and Data Acquisition

Antibodies used for mass cytometry are listed in Supplementary Tables 1, 2. Primary metal-conjugated antibodies were purchased from Fluidigm or purified antibodies were conjugated with metal reporters by using a MaxPar X8 Antibody Labeling kit (Fluidigm) according to manufacturer’s instructions. After conjugation, antibodies were diluted to 200 μl in antibody stabilization buffer (Candor Biosciences), supplemented with 0.05% sodium azide. Both antibody panels have previously been validated (24, 25), and in this study tested on both peripheral blood and decidual samples. Antibody staining and data acquisition were carried out as previously described (26, 27). In short, cells from decidual and peripheral blood samples were incubated with 1 mL of 1:500 diluted 500 μM Cell-ID Intercalator-^103^Rh (Fluidigm) for 15 min at room temperature (RT), washed, and incubated with human Fc blocking antibody (Biolegend) for 10 min at RT. Cell suspensions were thereafter stained with a mix of metal-conjugated antibodies for 45 min at RT. After washing, cells were incubated with 125 nM Cell-ID Intercalator-Ir (Fluidigm) in MaxPar Fix and Perm buffer (Fluidigm) and left overnight at 4°C. Prior to data acquisition, cell pellets were diluted in distilled water containing 1:10 diluted EQ Four Element Calibration Beads (Fluidigm), and cells were acquired by a Helios mass cytometer (Fluidigm). After acquisition, data was normalized using the EQ beads with passport P13H2302 reference. To account for technical variation, a PBMC reference sample from a healthy donor was included for both the general and the T cell panel at ten intervals during 20 staining batches and 18 CyTOF acquisition runs over a time period of 7 months.

### Mass Cytometry Data Analysis

For each data file, live single CD45+ immune cells were selected by gating in Cytobank (Supplementary Figure S1A). The gating strategy utilized the parameters residual, event length, width, and center to gate out debris and doublets. In addition, dead cells and normalization beads were excluded. Next, the files were subjected to sample-tagging, hyperbolic-arcsinh-transformation with cofactor 5 and dimensionality reduction in Cytosplore (28). Pair-wise Jensen-Shannon (JS) divergences were calculated for the individual samples within each tissue group, analyzed in a collective t-SNE, where low JS distances were indicative of high similarities between the samples within a group.

**FIGURE 1:**
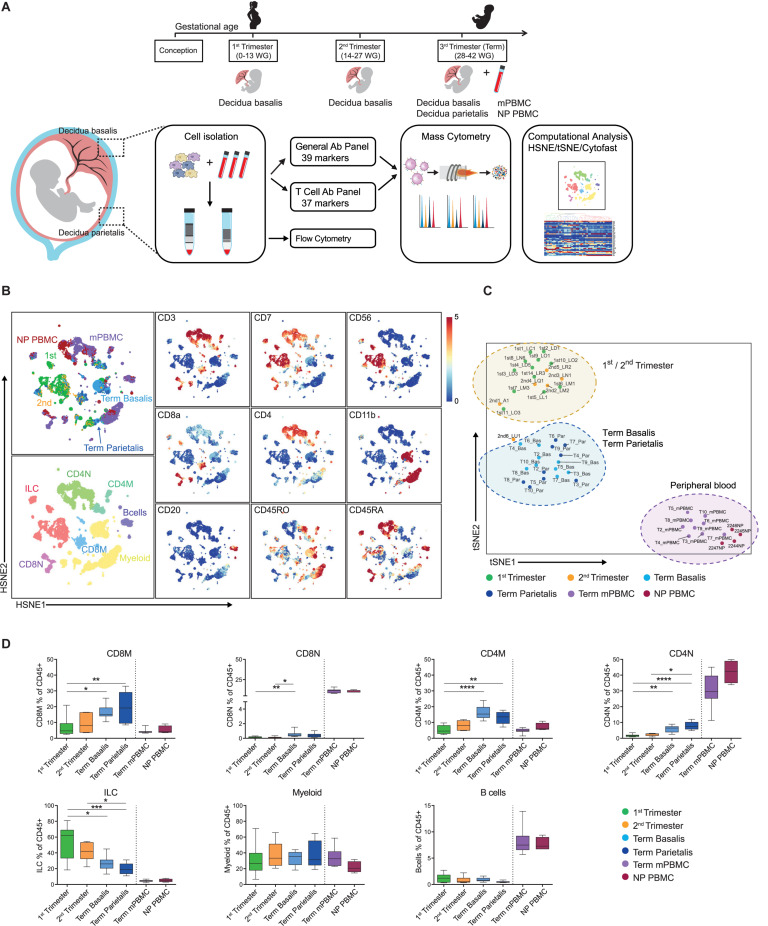
Identification of major immune cell lineages at the maternal-fetal interface. **(A)** Experimental setup. First (6–13 weeks of gestation), second (14–18 weeks) and third trimester (term; >38 weeks; basalis and parietalis) decidual samples along with maternal peripheral blood mononuclear cells (mPBMC) and non-pregnant PBMC (NP PBMC) were analyzed. **(B)** First-level HSNE visualization of the major immune cell lineages derived from decidua and peripheral blood. Colors top left indicate tissue type (1st trimester *n* = 12; 2nd trimester *n* = 6; term basalis and parietalis *n* = 9; mPBMC *n* = 9; NP PBMC *n* = 4); colors bottom left indicate major immune cell types (CD8M, CD8 memory T cells; CD8N, CD8 naïve T cells; CD4M, CD4 memory T cells; CD4N, CD4 naïve T cells; ILC, innate lymphoid cells); colors for plots on the right indicate the arcSinh5-transformed expression values of the specified markers where every dot represents a landmark. Memory and naïve clusters were distinguished based on CD45RO and CD45RA expression. **(C)** t-SNE visualization of the separation between decidual and peripheral blood samples (as percentage of CD45+ cells). Every dot represents a single sample. **(D)** Major immune cell lineages (as percentage of CD45+ cells) throughout gestation and within mPBMC and NP PBMC. Boxplots depict the 10–90 percentile and the Kruskal-Wallis with Dunn’s test for multiple comparisons was applied. *P < 0.05; **P < 0.01; ***P < 0.001; ****P < 0.0001.

All data were pooled per panel and a five-level HSNE analysis was performed with default parameters (perplexity 30; iterations 1,000), where the major immune cell lineages were identified by automatic clustering (Figure 1B and Supplementary Figures S1D, S2C). No influence of the mode of delivery on clustering of term decidual samples was observed in our analyses and a previous report by Tilburgs et al. (29) similarly confirmed no influence of mode of delivery and other clinical variables on decidual cell types in term pregnancy. All HSNE, t-SNE, and Gaussian mean-shift clustering-derived cell clusters were generated in Cytosplore. A cluster is defined as a population of at least 100 cells with the same phenotype. Exported FCS files for all identified individual clusters were subjected to the CytoFast workflow in R (30). Hierarchical clustering of the heatmaps was created with Euclidean correction and average linkage, and the median intensity values of markers were visualized. The number of cells in each immune cluster were determined for each sample and cluster frequencies and sample frequencies were calculated. Sample frequencies were visualized in boxplots and sample t-SNE plots. Violin plots, PCA plots and correlation network analysis were generated in R. Diffusion maps were generated in R using the “destiny” package (31). Within the CD4+ T cell compartment, CD4+ T_*N*_ cells together with the CD4+ T_*RORA*_ cluster and Treg-like T cell clusters branched off completely and were omitted from the final CD4+ T cell diffusion map. Within the CD8+ T cell compartment, CD27- CD69-, CD27+CD69- T_*N*_, and CD27^*INT*^CD69^*INT*^CD127+CCR6+ clusters branched off completely and were omitted from the final diffusion map. For the global test, incorporated within the Cytofast workflow, the absolute correlation distance with average linkage for hierarchical clustering was used. The branches colored in black show the significant multiplicity-corrected *p*-values.

### Flow Cytometry

Antibodies for flow cytometric analysis are listed in Supplementary Table 3. For surface staining, cells were stained for 30 min at 4°C in PBS 1% FCS. For intracellular staining, cells were fixed and permeabilized using the FOXP3 staining buffer kit (eBioscience). Acquisition and analysis were performed on an LSR-II (BD Biosciences) using FACS Diva software. In addition, HSNE and t-SNE analysis of flow cytometric data was performed using Cytosplore. Co-expression of FOXP3, HELIOS, CTLA-4, CD39, ICOS, and TIGIT was confirmed by manual gating and HSNE analysis.

### Statistical Analyses

Results are shown as median with interquartile range and the boxplots depict the 10–90 percentile. To determine differences among more than two unpaired groups, a non-parametric Kruskal-Wallis test with Dunn’s multiple comparison post-test was applied where significance was assessed by controlling for false discovery at 5% (FDR). *P*-values < 0.05 were considered to denote statistically significant differences. Statistical analyses were performed in GraphPad Prism version 8.0 and R version 3.5.1.

## Results

### The Maternal-Fetal Interface Harbors a Unique Immune Cell Composition

We analyzed first, second, and third trimester decidual samples along with mPBMC taken at the time of delivery and PBMC of non-pregnant age-matched women (NP PBMC) as a control (Table 1). A general mass cytometry panel comprising 39 antibodies (Supplementary Table 1) was used to provide a broad coverage of the myeloid and lymphoid immune compartments. For in-depth profiling of the T cell compartment, a second panel comprising 37 antibodies (Supplementary Table 2) was applied. After data acquisition (Table 2), live, single CD45+ cells were selected for downstream analysis (Supplementary Figure S1A and Figure 1A). Conventional cell populations were verified by manual gating and have previously been validated (27). To allow systematic comparison of samples, the data obtained with the general panel (49 samples; 19 × 10^6^ CD45+ cells) and the data obtained with the T cell panel (44 samples; 17 × 10^6^ CD45+ cells) were pooled separately and analyzed with hierarchical stochastic neighbor embedding (HSNE) and t-distributed stochastic neighbour embedding (t-SNE) in Cytosplore (28, 32). Comparison of the absolute numbers and percentages of CD45+ cells and correlations thereof showed a similar pattern in the general and T cell panel (Supplementary Figures S1B,C).

**TABLE 2:** Total number of cells and samples analyzed.

**Panel**	**# of Decidual samples**	**CD4+ T cells**	**CD8+ T cells**	**B cells**	**Myeloid cells**	**ILC/NK cells**	**TCRγδ cells**
General panel	36	1,136,799	1,082,234	72,414	2,390,451	3,841,125	114,875
T cell panel	32	818,800	707,147	73,579	2,087,932	3,556,369	96,640
Trimester	Total # of samples	General panel	T cell panel	Overlap (#, %)			
1st Basalis	14	12	11	9; 64%			
2nd Basalis	7	6	5	4; 57%			
3rd Basalis	9	9	8	8; 89%			
3rd Parietalis	9	9	8	8; 89%			
mPBMC	9	9	8	8; 89%			
NP PBMC	4	4	4	4; 100%			

At several timepoints during the acquisition timeline a PBMC reference sample was included, which corroborated reproducible staining and acquisition among different sets of experiments (Supplementary Figures S2A,B). Clustering of technical PBMC reference samples together with the experimental decidual samples (for each panel separately) using Cytosplore revealed that reference samples clustered tightly together and that variation between decidual samples was much greater than between reference samples (Supplementary Figures S3, S4). This demonstrated that only a limited amount of variation is explained by staining inconsistencies between batches.

At the overview level, the HSNE landmarks depicted the global data heterogeneity and marker expression profiles in both panels and identified the major immune cell subsets of myeloid cells, ILC, CD4+ T cells, CD8+ T cells (including the TCRγδ lineage), and B cells (Figure 1B and Supplementary Figure S1D). Subsequently, t-SNE analysis based on cell frequencies separated the samples of 1st and 2nd trimester from samples of term basalis and parietalis, and peripheral blood, indicative of distinct immune profiles (Figure 1C and Supplementary Figure S1E). Cell frequencies of the major immune cell lineages confirmed ILC as being the predominant cell type in 1st trimester, decreasing toward the end of pregnancy, and contrasting the dynamics of T cells. This analysis also validated that the number of myeloid cells remains relatively constant throughout gestation, while B cells are hardly present (Figure 1D and Supplementary Figure S1F) (6).

### Early Pregnancy Reveals a Heterogeneous Group of Myeloid Cells With High HLA-DR Expression

**FIGURE 2:**
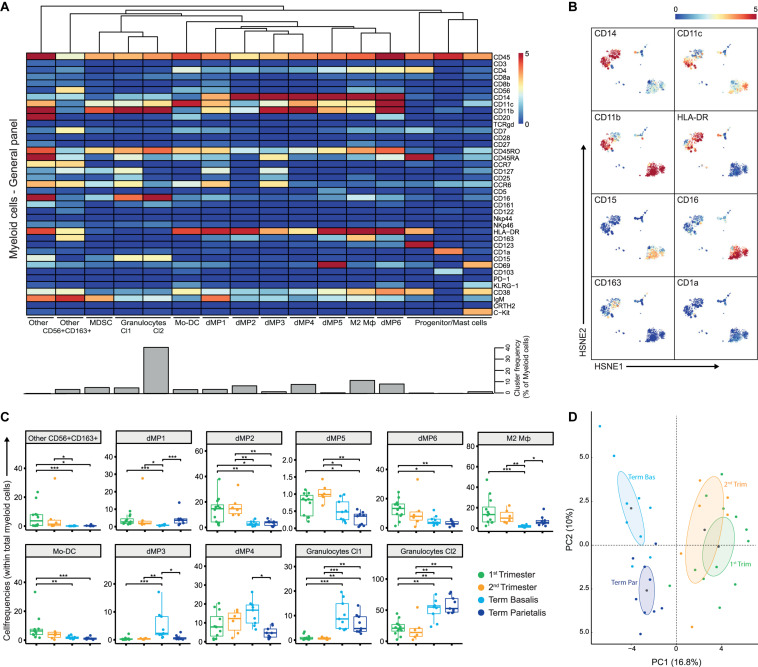
The myeloid compartment is highly diverse in early pregnancy. **(A)** Heatmap showing the median arcSinh5-transformed marker expression values for the 16 identified myeloid clusters within the general panel (36 samples; 2,390,451 cells). Cluster IDs and cluster frequencies are displayed at the bottom of the heatmap. **(B)** First-level HSNE embedding of the arcSinh5-transformed expression values of the indicated markers (note these are the same plots as in Supplementary Figure S2E). **(C)** Boxplots of sample frequencies, divided per trimester, of the cell clusters plotted as a fraction of total myeloid cells. The Kruskal-Wallis with Dunn’s test for multiple comparisons was performed and only clusters with significant differences (false discovery rate (FDR) < 5%) between the groups are shown. **(D)** Unsupervised principal component analysis (PCA) of the sample frequencies (as percentage of total myeloid cells), where the gestational age groups are depicted along the first two components. The centroid of each group is indicated in gray. MDSC, myeloid-derived suppressor cells; dMP, decidual mononuclear phagocytes; Mφ, macrophage. *P < 0.05; **P < 0.01; ***P < 0.001.

Next, for each antibody panel, the data from all decidual samples were pooled and HSNE analysis was performed on every lineage individually. Within the myeloid cell lineage (Supplementary Figure S2C), the second hierarchical level revealed six large subpopulations that could be discriminated based on differential expression of CD14, CD11c, CD11b, HLA-DR, CD16, and CD15 (Supplementary Figures S2D,E). Subsequently, Gaussian mean-shift clustering was applied and quantified with Cytofast (30), revealing 16 phenotypically distinct myeloid cell clusters (Figure 2A). Here, HSNE overview plots showed the individual markers that contributed to the separation into distinct clusters (Figure 2B). Next, we determined which myeloid cell clusters were differentially present in 1st and 2nd trimester, term basalis, and term parietalis samples to uncover dynamics throughout pregnancy (Figure 2C). Only cell clusters with significant differences (false discovery rate (FDR) <5%) between the groups are shown.

Notably, early pregnancy (1st and 2nd trimester) was characterized by the presence of a heterogeneous group of myeloid cells with high HLA-DR expression. CD163+HLA-DR+ cells, also expressing intermediate levels of CD56 and CD7 (cluster “Other CD56+CD163+”), were observed in the 1st and 2nd trimester (Figure 2C), and may represent myeloid-like NK cell progenitors or a distinct monocyte/dendritic cell population. Furthermore, cell clusters of decidual mononuclear phagocytes (dMP), namely dMP1, dMP2, dMP5, and dMP6, expressing various combinations of CD14, CD11b, CD11c, CCR6, CD38, and CD69 were more prominent in 1st and 2nd trimester decidua compared to term decidua. The immune-regulatory CD163+ M2 macrophage (Mφ) subtype was present in early pregnancy and term decidua parietalis, but hardly in term decidua basalis. In addition, CD11c^*high*^CD14-CD16- Mo-DC were predominantly abundant in 1st trimester. Moreover, CCR6+CD45RA+CD38- dMP cell clusters with low HLA-DR expression (dMP3 and dMP4) were dominantly present in term decidua basalis while the largest population of CD15+CD16+ granulocytes was found in both term decidua basalis and parietalis (Figure 2C). Finally, a clear separation between early and late pregnancy samples in unsupervised principal component analysis (PCA; Figure 2D) was driven by an abundance of granulocytes in late pregnancy and supported by a previously unrecognized diverse composition of myeloid cells in early pregnancy. Together, these results reveal substantial changes in the composition of the myeloid compartment during gestation.

### Dynamic Changes in the Composition of the ILC Compartment During Pregnancy

A similar analysis of the ILC compartment (CD3-CD7+) confirmed its well-described cellular composition in decidua (33–36). The general panel classified 14 clusters with high expression of CD56 and lack of CD16 (Supplementary Figure S5A). Early pregnancy was characterized by activated CD161+CD122+NKp46+CD69+ NK cells (NK2, NK3, NK5, NK7, NK8, NK13), tissue-resident CD69+CD103+ cell clusters (NK4, NK5, K7) and ILC3 (Supplementary Figures S5A,B), coupled to the expression of CD39 and TIM-3 (Supplementary Figures S5C–E) (37). Toward the end of pregnancy, NK cells displayed a less activated phenotype with lower expression of CD161, CD122, NKp46, and CD103, and higher expression of CD45RA and CD16 (mostly in term basalis; Supplementary Figure S5B). Tissue-resident-like ILC were not only observed in 1st trimester (dIC6; decidual ILC Cluster), but also in small numbers in term samples (dIC1) along with the expression of TIGIT (Supplementary Figures S5C,D). In addition, expression of the co-inhibitory receptors TIM-3 and CD39 was observed in both early and term parietalis samples. NK2, NK3, and NK5 clusters resembled a phenotype similar to the intermediate innate subset described in fetal intestine that can differentiate into ILC3 and NK cells (25).

In summary, high proportions of activated ILC are present early in pregnancy alternated by dissimilar, smaller proportions of ILC cell clusters in term pregnancy, where the largest separation was observed between 1st trimester and term basalis (Supplementary Figure S5F).

### The Decidua Harbors NKT-Like TCRγδ T Cells

Substantial phenotypic diversity was observed within decidual TCRγδ cells where seven cell clusters were identified within the general panel (Supplementary Figures S6A,B). The most prominent cell clusters were CD161+KLRG1+ TCRγδ_*EM*,_ present throughout gestation, and CD69+ TCRγδ_*EMRA*_ that were dominant in term basalis. Remarkably, NKT-like populations of TCRγδ cells expressing CD56 and CD11c were also observed. T_*EMRA*_, with high expression of CD45RA, and T_*EM*_ cell clusters persisted in early pregnancy while cells co-expressing CD45RA and CD45RO and positive for CD27, CD5, and CD69 increased in term parietalis (Supplementary Figures S6A,C). Even though differences throughout gestation were existent, close clustering between the three different trimesters was observed in a PCA (Supplementary Figure S6D). In summary, these results display heterogeneity and the presence of NKT-like populations within the TCRγδ compartment.

### CD4+ T Cell Characterization Reveals Unexplored Diversity Within Memory and Regulatory Phenotypes

**FIGURE 3:**
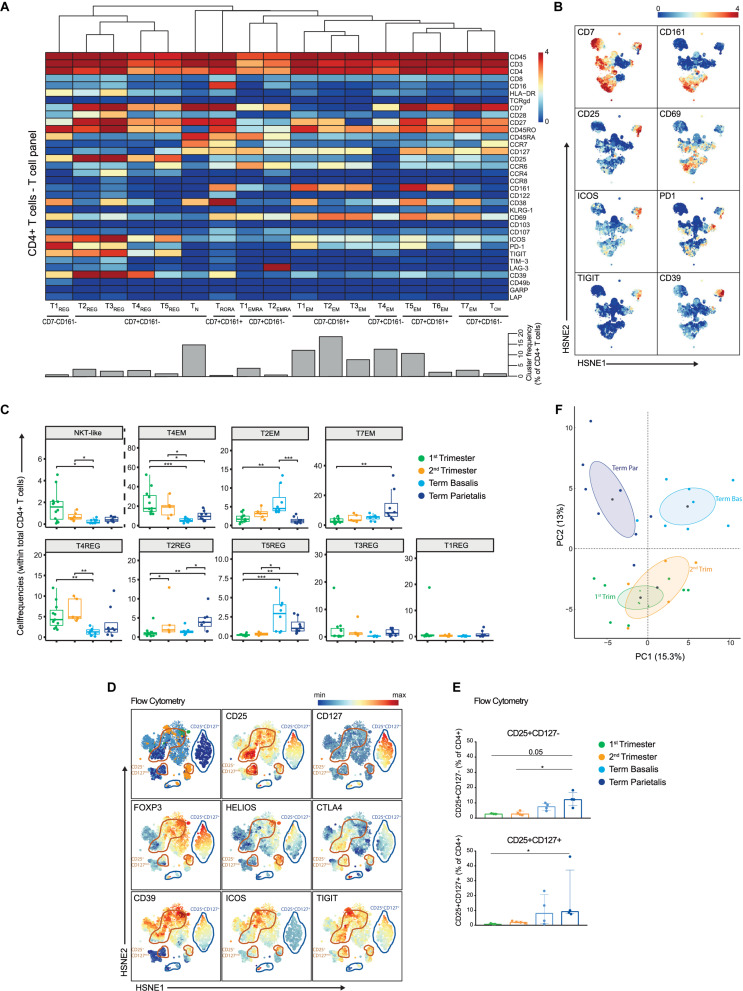
In-depth characterization of the heterogeneity within the CD4+ T cell compartment. **(A)** Heatmap showing the marker expression values for the 17 identified CD4+ T cell clusters within the T cell panel (32 samples; 818,800 cells). Cluster IDs and cluster frequencies are displayed at the bottom of the heatmap. **(B)** First-level HSNE embedding of the expression values of the indicated markers. **(C)** Boxplots of sample frequencies, divided per trimester, of the clusters plotted as a fraction of total CD4+ T cells. The Kruskal-Wallis with Dunn’s test for multiple comparisons was performed. **(D)** HSNE embedding of the expression values of the indicated markers, measured by flow cytometry and gated within CD3+CD4+ T cells. CD4+CD25+CD127- clusters are circled in orange; CD4+CD25+CD127+ clusters are circled in blue. 1st (*n* = 3), 2nd (*n* = 4) and term decidua (*n* = 4). **(E)** Boxplots depicting the CD25+CD127- (upper panel) and CD25+CD127+ (lower panel) populations as percentage of CD4+ T cells. **(F)** PCA of the sample frequencies (as percentage of total CD4+ T cells) where the gestational age groups are depicted along the first two components. The centroid of each group is indicated in gray. *P ≤ 0.05; **P ≤ 0.01; ***P ≤ 0.005.

In the CD4+ T cell lineage 17 cell clusters were identified: one naïve (N; CD45RA+CCR7+), two terminally differentiated (TEMRA; CD45RA+CCR7−), one central-memory (CM; CD45RO+CCR7+), seven effector-memory (EM; CD45RO+CCR7−), one CD45RA+RO+ and five memory regulatory-like T cell (Treg-like; CD25+CD127−) clusters (Figures 3A,B). Early in pregnancy, natural-killer-like CD4+ T cells (NKT-like) exist that express CD56, CD11c, CD161, CD122, NKp46, and CD38 (Supplementary Figure S7A and Figure 3C). Expression of CD127 and CCR6 occurred toward the end of pregnancy (T2_*EM*_), consistent with the early pregnancy-associated T4_*EM*_ cluster that lacked expression of these markers. At term, CD4+CD7-CD161+ T_*EM*_ cells expressing CD27 and CCR6 (T2_*EM*_) were observed in term basalis, whereas CD4+CD7+CD161- T_*EM*_ cells expressing CD38 and ICOS, and lacking CCR6 (T7_*EM*_) were predominantly present in term parietalis (Figure 3C). Furthermore, CD4+ T_*EM*_ cells showed co-expression of PD-1 and ICOS, at lower levels than the Treg-like population, and lack of TIGIT and CD39.

Considerable heterogeneity within the Treg-like compartment was uncovered, where CD25+CD127- cell clusters expressed high levels of co-inhibitory (PD-1, CD39, TIGIT) and stimulatory (ICOS, CD38, CD28, CD27) receptors, including co-expression thereof (Figures 3A,B). When investigating the Treg-like compartment in more detail, previously unrecognized heterogeneity was observed with respect to the expression of the Treg-associated markers TIM-3, CCR8, and CCR4 (Supplementary Figure S7B) (38–40). Tr1 cells, identified by co-expression of LAG-3 and CD49b (41), were absent in decidual CD4+ T cells (Supplementary Figure S7B), but were observed in mPBMC (data not shown). Quantification of the presence of these CD25+ cell clusters in the gestational age groups revealed that T4_*REG*_ (HLA-DR-CD69-PD-1-) and T3_*REG*_ (CCR4+CD38+) were more frequent in early pregnancy and lower in term basalis, whereas the largest Treg-like population, T2_*REG*_ (ICOS+PD-1+TIGIT+CD39+), was significantly increased in term parietalis (Figure 3C). Furthermore, T5_*REG*_ (CCR6+ICOS+TIGIT+PD-1-CD39-) was significantly increased in term decidua basalis and parietalis, while virtually absent in early pregnancy. By aligning cells from these five Treg-like clusters along a two-dimensional diffusion map (31), putative differentiation and/or plasticity trajectories were observed between cell clusters T2_*REG*_, T3_*REG*_, T4_*REG*_, and T5_*REG*_. T1_*REG*_, the smallest Treg-like cluster, was distinct owing to the lack of CD7 and CD27 expression (Supplementary Figure S7C).

To further evaluate the Treg-like phenotypes, intracellular expression of FOXP3, HELIOS, and CTLA-4 in CD4+CD25+CD127- and CD127+ T cells was assessed by flow cytometry in decidual samples (Supplementary Table 3). Co-expression of FOXP3, HELIOS, CTLA-4, CD39, ICOS, and TIGIT was observed in HSNE analysis of flow cytometry data, confirming a valid regulatory T cell phenotype (Figure 3D). In addition, differential co-expression of these markers was observed in several cell clusters, where not all CD4+CD25+CD39+ICOS+ cells expressed FOXP3 and/or HELIOS. This indicates that the Treg-like CD25+CD127- populations detected by mass cytometry represent a heterogeneous group of Treg and Treg-like cells at the maternal-fetal interface (Figure 3D). Flow cytometry data revealed an increase in CD4+CD25+CD127+ T cells, known to be activated effector CD4+ T cells (42), and regulatory-like CD4+CD25+CD127- T cells toward the end of pregnancy, with this increase being most apparent in term parietalis (Figure 3E and Supplementary Figure S7D). Overall, the data uncovered distinct memory and regulatory-like CD4+ T cell populations at different locations throughout pregnancy, where clear separation is revealed between early and term pregnancy, as well as between term basalis and parietalis (Figure 3F).

**FIGURE 4:**
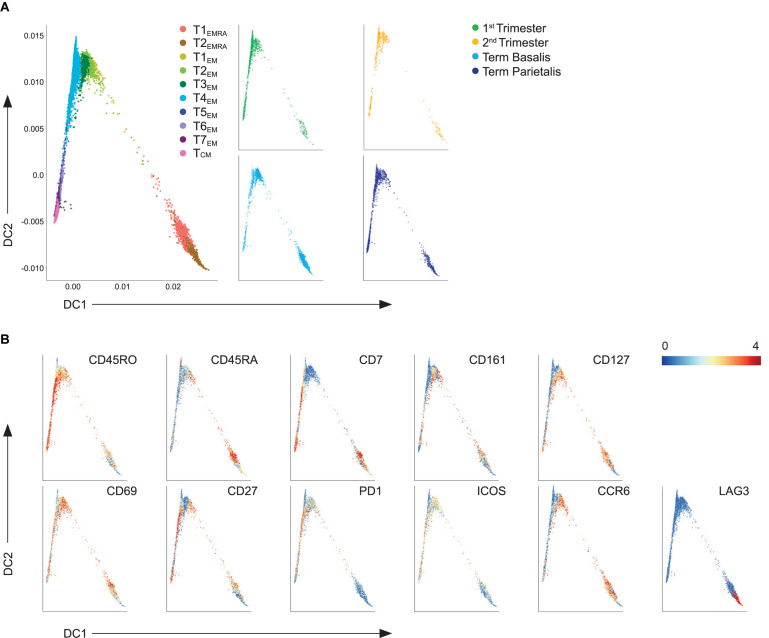
Trajectory analysis of effector and memory decidual CD4+ T cells. **(A)** Visualization of terminally differentiated (EMRA) and effector-memory (EM) CD4+ T cell clusters in a diffusion map along two components. Each color in the left panel represents a cluster of cells. In the right panel, cells within the 1st trimester, 2nd trimester and term decidua basalis and parietalis are portrayed. **(B)** ArcSinh5-transformed expression values of the specified markers in the diffusion map.

Next, diffusion mapping was used to distinguish prospective relationships among the different types of memory CD4+ T cell clusters. Two-dimensional diffusion plots revealed a split into two branches with T4_*EM*_, lacking CD127 expression, at the center of the split (Figure 4A). Gradients of protein expression between cells were observed rather than discrete cell clusters (Figure 4B). The branch that expanded along diffusion component 2 (DC2) consisted of CD7+CD161+ and CD161- T_*EM*_ cells that were CD127+ and CD27+. T_*CM*_ was projected at the end of this trajectory branch. The second branch along DC1 consisted of the CD7-CD161+ clusters that showed CD127 expression, including one cluster (T3_*EM*_) that lacked CD27 expression. The two EMRA clusters separated out from the EM clusters based on their expression of CD45RA and lack of CD27 expression. These results suggest putative differentiation states between the identified EM CD4+ T cell clusters throughout pregnancy.

### Decidual CD8+ T Cells Co-express Inhibitory and Stimulatory Receptors

**FIGURE 5:**
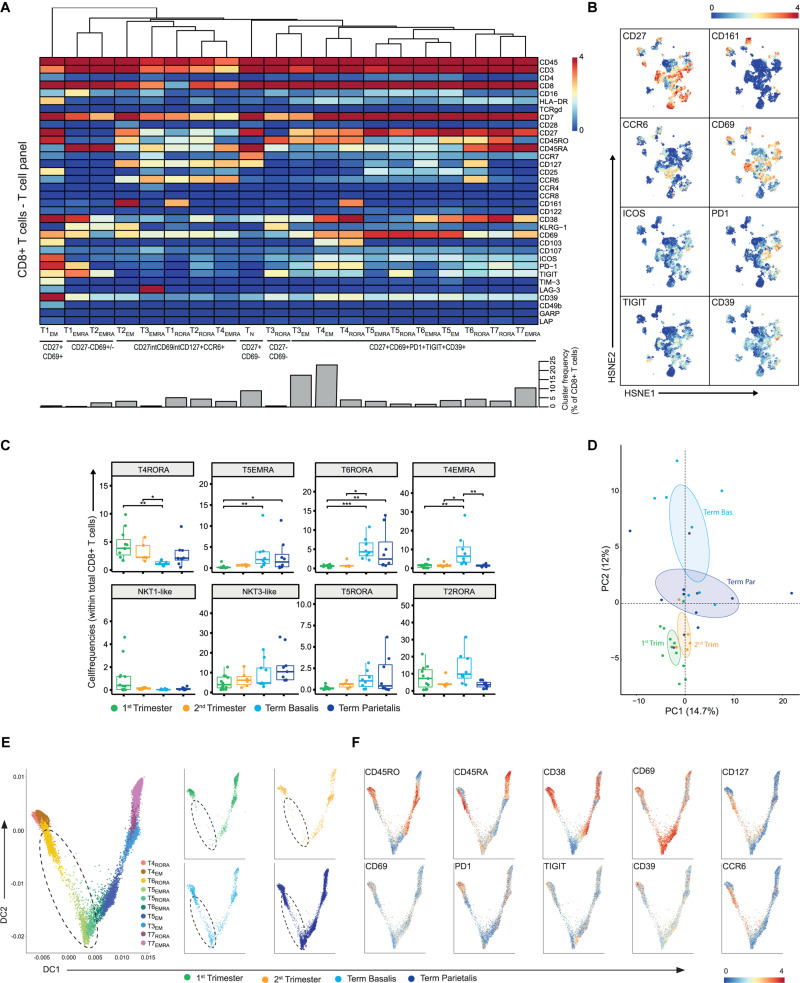
In-depth characterization of the heterogeneity within the CD8+ T cell compartment. **(A)** Heatmap showing the marker expression values for the 20 identified CD8+ T cell clusters within the T cell panel (32 samples; 707,147 cells). Cluster IDs and cluster frequencies are displayed at the bottom of the heatmap. **(B)** First-level HSNE embedding of the expression values of the indicated markers. **(C)** Boxplots of sample frequencies, divided per trimester, of the clusters plotted as a fraction of total CD8+ T cells. The Kruskal-Wallis with Dunn’s test for multiple comparisons was performed. **(D)** PCA of the sample frequencies (as percentage of total CD8+ T cells) where the gestational age groups are depicted along the first two components. The centroid of each group is indicated in gray. **(E)** Visualization of T_*EMRA*_, T_*RORA*_, and T_*EM*_ CD8+ T cell clusters in a diffusion map along two components. Each color in the left panel represents a cluster of cells. In the right panel, cells within the 1st trimester, 2nd trimester and term decidua basalis and parietalis are portrayed. **(F)** ArcSinh5-transformed expression values of the specified markers in the diffusion map. *P ≤ 0.05; **P ≤ 0.01; ***P ≤ 0.005.

We next investigated the heterogeneity within the CD8+ T cell compartment where 20 CD8+ T cell clusters were characterized, namely one naïve, seven TEMRA, five EM, and seven clusters co-expressing CD45RA and CD45RO, a phenotype that is associated with proliferation (Figures 5A,B). Four of these clusters revealed significant differences between the decidual samples (Figure 5C). The tissue-resident memory (TRM) CD8+ T cell cluster T4_*RORA*_ (CD69+CD103+CD38+CD161+PD-1+CD39+) was more frequent in early pregnancy, while T5_*EMRA*_ (CD69^*high*^) and T6_*RORA*_ (CD127+CCR6+CD38+CD69+) were more abundant in term samples. Also, T4_*EMRA*_ (CD127+CCR6+) was increased in term basalis. In addition, a trend for a higher presence of NKT1-like cells in the 1st trimester, a gradual increase in NKT3-like and T5_*RORA*_ cells from 1st trimester to term, and higher numbers of T2_*RORA*_ in 1st trimester and term basalis were observed (Figure 5C and Supplementary Figure S8A). High levels of CD27 were observed in several effector and effector-memory cell clusters (e.g., T5_*EMRA*_, T5_*RORA*_, T6_*RORA*_).

Where our recent work demonstrated a mixed gene expression signature of activation and dysfunction in bulk memory decidual CD8+ T cells (43), mass cytometry at the single-cell level revealed the expression of inhibitory and stimulatory receptors to be intertwined (Figure 5A). This co-expression of inhibitory (CD39, PD-1, TIGIT) and stimulatory (ICOS, CD69, CD27) receptors was verified by flow cytometry and mainly observed in term basalis and parietalis (Supplementary Figure S8B). Interestingly, the T_*EMRA*_ and T_*CM*_ clusters within the CD8+ T cell compartment contrasted the frequencies of these populations within the CD4+ T cell compartment with a higher percentage of T_*EMRA*_ and lower percentage of T_*CM*_ within the CD8+ T cells (Supplementary Figure S8C). In general, the differences in marker expression in the CD8+ T cell compartment were more subtle when compared to the CD4+ T cells. Consequently, the PCA showed a less clear separation between early and late pregnancy with term parietalis being more similar to 1st and 2nd trimester samples than term basalis (Figure 5D).

In a two-dimensional diffusion plot analysis, two branches were observed with the CD38- clusters (T5_*EMRA*_, T5_*RORA*_) at the center of the split. Here, the CD38+CD69+ T_*EMRA*_ and T_*EM*_ clusters expanded along DC1, while the TRM cells and CD127+CCR6+ T_*EM*_ cells expanded along DC2 (Figures 5E,F). Furthermore, along DC2 cell clusters T5_*EMRA*_, T5_*RORA*_, and T6_*RORA*_ with lower expression of CD45RO and PD-1 and high expression of CD69, were absent in early pregnancy and appeared in term pregnancy, as observed in Figure 5C (Figure 5E; dashed circle). These potential differentiation trajectories suggest a phenotypic continuum and thereby possible plasticity between specific CD8+ T cell clusters.

In summary, these data show that a group of CD8+ T cells displays co-expression of inhibitory and stimulatory receptors at the protein level, and suggest that in this group several differentiation trajectories coupled to distinct functions throughout gestation may be at play. Whereas CD8+ NKT cells are present in early and late pregnancy, there are hardly any CM CD8+ T cells.

### B Cells Are Mainly Present Early in Pregnancy

Although the number of B cells was low, nine CD20+ B cell clusters with variable expression of CD38, CD27, and IgM were identified within the general panel (Supplementary Figure S9A). Interestingly, most B cells were detected in the 1st trimester (Supplementary Figure S9B). CD20 was also included in the T cell panel (as exclusion marker) and showed to be useful in detecting CD39 expression on several B cell clusters (Supplementary Figure S9C).

### Correlation Analysis Reveals Gestational-Specific Immune Networks

**FIGURE 6:**
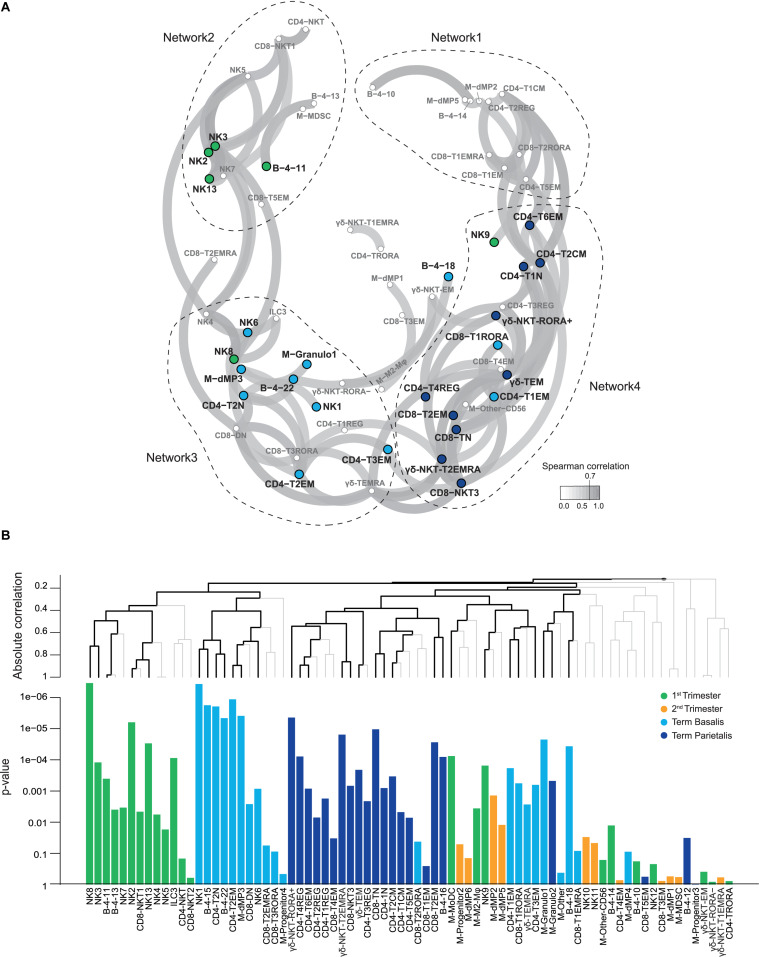
Relationships between identified cell clusters from all major immune cell lineages. **(A)** Correlation network plot showing Spearman coefficients higher than 0.7 for relationships between the decidual immune cell clusters from the general panel. Every circle depicts an immune cell cluster, with colors corresponding to statistically significant contributions of clusters to 1st trimester, term decidua basalis or parietalis, as calculated by the global test (44). Higher correlation corresponds to greater proximity of cell clusters. **(B)** Multivariate associations between cell clusters detected by a multinomial logistic regression model with the global test. The top panel shows hierarchical clustering of absolute correlation distances, where the branches in black indicate significant multiplicity-corrected *p*-values. The bottom panel shows the corresponding *p*-values of immune cell clusters associated with 1st trimester (green), 2nd trimester (yellow), term decidua basalis (light blue), and parietalis (dark blue).

To conflate the 77 identified immune cell clusters within the general panel and visualize relationships between them, a correlation network analysis was performed using the sample frequencies. This analysis demonstrated that 73% of clusters were strongly correlated with each other (Spearman rank >0.7; Figure 6A). Subsequently, multivariate associations between individual and groups of clusters were detected by applying a multinomial logistic regression model with the global test (Figure 6B) (30, 44). Four networks were revealed in which colored nodes highlight the significance of individual cell clusters in one of the four gestational age groups. Cell clusters in network 1 consisted of myeloid cells, CD4+ T cells, CD8+ T cells, and B cells, and did not reveal significant gestational specificity. Network 2 revealed a correlation between NKT-like, B cell, and NK cell clusters (including tissue-resident-like phenotypes), most of which were significantly abundant in the 1st trimester. This may reflect unappreciated interactions between NK cells and NKT-like cells early in pregnancy. Network 3 is characterized by clusters predominantly present in term basalis and included a correlation between innate immune cells such as NK cell clusters, dMP3 and granulocytes, and adaptive immune cells including T_*N*_, T_*EM*_ and Treg-like CD4+ clusters, CD8+ T_*RORA*_ cells, and TCRγδ_*EMRA*_ cells. Interestingly, a different network of clusters was observed in term parietalis (network 4), where CD4+ and CD8+ T_*N*_ cells, CD4+CD127+CD161- T_*CM*_ cells, CD4+ Treg-like clusters, CD8+ T_*EM*_ and NKT-like cells, and TCRγδ cells were correlated. These results underline that distinct immune cell interactions in basalis versus parietalis contribute to the microenvironment in term pregnancy. Thus, three of the four networks correlated with either gestational age or tissue location.

## Discussion

To better understand the maternal immune landscape during healthy pregnancy, we performed mass cytometry analysis of immune cells isolated from decidua throughout the three trimesters of pregnancy and compared this to term mPBMC. This provided an unbiased, data-driven overview of all decidual immune cell populations throughout pregnancy. Previously described decidual immune cell subsets (5, 33, 45, 46) and the kinetics of the major immune cell lineages during gestation (5, 6, 47) were validated in the current study. Moreover, we observed unprecedented immune cell heterogeneity in the decidua.

By implementing replicate PBMC control samples along with the experimental decidual samples, we demonstrated that the identified decidual immune cell clusters described here displayed much greater phenotypic diversity than what could be explained by staining inconsistencies and that batch effects are therefore minimal. It should be noted that 11 (tissue-specific) out of 52 unique markers of both antibody panels combined displayed no or hardly any expression in the internal control PBMC reference samples and could, therefore, not be assessed for staining consistency during batch analysis.

Distinct clusters of dMP were detected in early pregnancy, suggestive of an essential role for antigen presentation and thereby interaction with other immune cells at the initiation of pregnancy. Furthermore, the presence of different dMP cell clusters in term basalis and parietalis may reflect distinct local antigen presentation and function. Proportions of the dMP cells decrease over gestation accompanied by an influx of granulocytes at time of parturition, in line with the observed increase in the numbers of circulating neutrophils during pregnancy (48). ILC that play an important role in early pregnancy by facilitating spiral artery remodeling and trophoblast invasion, may in small proportions preserve their function (e.g., play a role in the clearance of infections) in term pregnancy where they display a less activated phenotype with the expression of inhibitory and tissue-residency receptors.

Most studies on decidual Treg have thus far focused on CD4+FOXP3+ T cells (16, 49, 50). Our present mass and flow cytometry data confirmed the presence of other, recently described, FOXP3^*low/–*^ decidual Treg subtypes (51). Furthermore, we observed additional heterogeneity, with co-expression of inhibitory and stimulatory receptors and clusters lacking expression of FOXP3 and/or HELIOS, revealing a mixed population of Treg and Treg-like cells. It supports the hypothesis that both natural (nTreg) and induced Treg (iTreg) play a role, where bright expression of CD25 is not a prerequisite for Treg function. A decrease in FOXP3 and HELIOS expression toward term suggests a decline of nTreg and increase of iTreg throughout gestation (51). These Treg populations are induced, among others, by EVT and decidual Mφ (51), and may therefore have distinct cellular targets, which likely include the formerly unexplored heterogeneous group of memory CD4+ T cells. Evidence exists that 1st trimester decidual CD4+ T cells have transcriptional profiles compatible with antigen-induced activation and proliferation (52). Moreover, decidual CD4+ T cells isolated from term decidua showed fetal antigen-specific responses that were enhanced upon depletion of CD25+CD127- Treg (12). Presence of paternal antigen-specific Treg in the decidua has been suggested (29), and clonal expansion of both decidual Treg (53) and memory CD4+ T cells by locally presented antigens is suggested by preliminary data from our laboratory showing a restricted CDR3 length distribution of the TCRVβ repertoire in term decidual CD4+ T cells compared to peripheral CD4+ T cells (data not shown). The observed increase in activated CD4+ T cells may be counteracted by an increase in Treg in term parietalis to secure success of pregnancy. Evidently, functional assays are necessary to further explore the co-existence of effector memory CD4+ T cells with nTreg and iTreg, especially in the context of complicated pregnancies (54). Treg may also be essential in the regulation of distinct CD4+ and CD8+ NKT-like clusters in early pregnancy, as suggested by the increased percentages of NKT cells observed in women with unexplained recurrent spontaneous abortions (55).

Recent research on fetal-specificity (12), virus-specificity (56), and cross-reactivity of decidual CD8+ T cells with HLA-C (57), complemented by the herein described co-expression of inhibitory and stimulatory receptors, emphasizes the dual role of CD8+ T cells in both tolerance and immunity. Co-expression of CD45RO/RA in several clusters hints at local proliferative potential, and interactions with APC and Treg may be essential to control CD8+ T cells at the maternal-fetal interface. Furthermore, recently addressed contributions of TCRγδ T cells to transplantation outcomes and their role in HIV controllers (58, 59) advocate for an unexplored functional role of TCRγδ T cell subsets in early and term pregnancy, which requires further exploration. B cell clusters expressing CD39, a marker involved in the activation of B cells to suppress T cells (60), might resemble regulatory B cells. Alterations in B cell function in early pregnancy has been suggested to play a role in recurrent miscarriages, where a higher incidence of anti-HLA-C antibodies was observed in women with recurrent miscarriage (61).

Diffusion mapping revealed putative differentiation trajectories of effector, memory, and regulatory T cells throughout gestation, emphasizing the dynamic state and conceivable plasticity of decidual T cells in response to environmental cues. It should be kept in mind, however, that the cell phenotype trajectories may partly be influenced by recruitment of immune cells into the tissue as gestation progresses. In both the CD4+ and CD8+ compartment an increase in activated effector T cell phenotypes toward the end of pregnancy suggests an inflammatory state required for parturition. Subsequently, combining all identified immune cell clusters in a correlation network analysis demonstrated that the local immune landscape as a whole, and not isolated cell subsets, develops as an integrated system throughout gestation. Co-expression of inhibitory and stimulatory receptors in this system is prominent and needs to be finely balanced to ensure a successful pregnancy. The prominent connection between myeloid cells and T cells (network 1) at any time point during pregnancy reflects their bi-directional interactions both in a contact-dependent manner and through cytokine excretion. The connection between NKT-like cells and NK cells specifically in the first trimester (network 2) needs further exploration. Differences in immune cell networks and their prospective functions observed between term basalis (network 3) and parietalis (network 4) suggest possible distinct antigen availability and presentation at these two placental locations. More regulatory phenotypes were observed in the parietalis with increased percentages of Treg, M2 Mφ, and TRM CD8+ T cells. This observation may be in line with findings of a single cell analysis of separate placental compartments (62), showing that the basal plate (including the basalis) contains more activated T cells and less resting T cells compared to the chorioamniotic membranes (including the parietalis). The abundant density of lymphatic vessels in the region adjacent to the chorionic membrane, which is attached to the parietalis, suggests that antigen presentation and activation need to be carefully controlled at this site (63). Term basalis consistently showed cell clusters with higher expression of CCR6, a receptor involved in chemotaxis. The influx of immune cells might therefore be more common in term basalis.

This study has its limitations. First, in human pregnancy studies the unavailability of uncomplicated decidual samples between 24 and 37 weeks of gestation results in a gap in our knowledge and understanding of the complete second trimester. Second, mass cytometry identifies phenotypic diversity based on preselected markers and provides little insight into the functionality of identified cell clusters. Here, we investigated the T cells in depth, but additional myeloid and B cell-specific markers are necessary to explore the complexity within these lineages. Although the rationale for defining a cluster is the presence of at least 100 cells with the same phenotype, further research needs to be performed to confirm if the identified subclusters represent true, functionally distinct, subpopulations. It is plausible that some of the phenotypically distinct cell clusters are differentiation stages between cell populations, as suggested by our diffusion mapping data. Therefore, the results from the current study should be considered as a basis for subsequent investigations. Future studies constituting a validation cohort with additional healthy decidual samples and including samples from complicated pregnancies will provide comprehensive insight into generalizable differences between healthy and complicated pregnancy. Although decidual and peripheral blood immune cells clustered completely separate in t-SNE analyses, trafficking of cells between these two entities almost certainly occurs (64, 65). In pregnancy complications both systems should be studied in parallel as the occurrence of certain cell subsets in the blood, possibly precursors, may predict what takes place locally in the decidua and thereby serve as biomarkers to predict complications.

In the field of reproductive immunology, a shift toward systems biology with a focus on interactions between cell types and away from studying isolated cell populations is required. This ecosystem where not only maternal immune cells but also EVT, decidual stromal cells, endothelial cells, and micro-organisms are coordinated with each other needs to be explored in more depth, and in relation to pregnancy complications presenting a more heterogeneous microenvironment than expected. In this context, single-cell RNA sequencing has revealed potential cell-cell interactions at the maternal-fetal interface (37). Future studies will benefit from combining mass cytometry data and RNA sequencing to cross-validate transcriptional activity and protein levels of singular cells, and from incorporating imaging CyTOF to define the cellular anatomical locations. Furthermore, the generation of trophoblast organoids as a model for maternal-fetal interactions (66), development of a placenta-on-a-chip (67), and interconnectivity analysis of multiple biological systems such as metabolomics and transcriptomics (37, 68) will further enhance our understanding of the placenta and the cellular interactions within this ecosystem.

Taken together, mass cytometry enabled us to visualize the complex and dynamic network of decidual immune cell populations at the maternal-fetal interface, where during uncomplicated pregnancy coordinated interaction is vital for a successful outcome. The immune atlas as presented here may serve as a foundation for further identification and functional analyses of immune subsets in healthy versus complicated pregnancies.

## Data Availability Statement

Mass cytometry data are available via Flow Repository (https://flowrepository.org/id/FR-FCM-Z3YF).

## Ethics Statement

The studies involving human participants were reviewed and approved by Medical Ethics Committee of the Leiden University Medical Center. The patients/participants provided their written informed consent to participate in this study.

## Author Contributions

AZ, VU, FC, and SH designed the research and with help of FK wrote the manuscript. AZ performed the experiments with help of SL, CK, and HK. AZ performed the data analyses with help of VU, GB, SL, and TH. Conceptual input was provided by VU, GB, ME, and FK. Clinical samples were provided by M-LH and SC. All authors contributed to finalizing the manuscript. All authors contributed to the article and approved the submitted version.

## Conflict of Interest

The authors declare that the research was conducted in the absence of any commercial or financial relationships that could be construed as a potential conflict of interest.

## Supplementary Material

FIGURE S1 Identification of major immune cell lineages at the maternal-fetal interface using the T cell panel. **(A)** Gating strategy to select single, live CD45+ cells for downstream analysis. **(B)** Comparison of the absolute numbers and percentages of CD45+ cells measured by the general and the T cell panel. **(C)** Correlation plots of CD45+ cells measured by the general and T cell panel within the three trimesters, maternal peripheral blood mononuclear cells (mPBMC) and non-pregnant control samples (NP PBMC). **(D)** First-level HSNE visualization of the major immune cell lineages derived from decidua and peripheral blood. Colors top left indicate tissue type (1st trimester *n* = 11; 2nd trimester *n* = 5; term basalis and parietalis *n* = 8; mPBMC *n* = 8; NP PBMC *n* = 4); colors bottom left indicate major immune cell types (CD8M, CD8 memory T cells; CD8N, CD8 naïve T cells; CD4M, CD4 memory T cells; CD4N, CD4 naïve T cells); colors for plots on the right indicate the arcSinh5-transformed expression values of the specified markers where every dot represents a landmark. Memory and naïve clusters were distinguished based on CD45RO and CD45RA expression. **(E)** t-SNE visualization of the separation between decidual and peripheral blood samples (as percentage of CD45+ cells); every dot represents a single sample. **(F)** Major immune cell lineages (as percentage of CD45+ cells) throughout gestation and within mPBMC and NP PBMC. Boxplots depict the 10–90 percentile and the Kruskal-Wallis with Dunn’s test for multiple comparisons was applied. ^∗^*P* < 0.05; ^∗∗^*P* < 0.01; ^∗∗∗^*P* < 0.001.

FIGURE S2 t-SNE visualization of PBMC reference samples and partitioning of the myeloid cell compartment into subpopulations. Cell frequencies (as percentage of CD45+ cells) are plotted where every dot represents a single sample within the general panel **(A)** and within the T cell panel **(B)**. The gray arrow indicates the PBMC reference control samples clustering together. **(C)** HSNE overview (first) level embedding of all decidual samples with identification of the major immune cell lineages based on lineage marker expression. **(D)** Second-level HSNE embedding of the myeloid cells subdivided into six major subpopulations. **(E)** Second-level HSNE arcSinh5-transformed expression values of the specified markers where every dot represents a landmark.

FIGURE S3 Analysis of staining fluctuations between batches for the general CyTOF antibody panel. Nine replicate control samples from the same blood donor stained with the general CyTOF panel measured throughout the 7-month study period. (A) A t-SNE embedding showing the collective CD45+ cells (14.5 × 10^4^ cells) from nine replicate control samples and 20 experimental decidual samples. Colored dots represent single cells from replicate samples and gray represents experimental samples. (B) Same t-SNE embedding as in panel A, colored for each replicate sample. (C) A t-SNE plot showing 25 cluster partitions in different colors. (D) Composition of the cell clusters in the individual samples (*n* = 29) represented in horizontal bars where the size of the colored segments represents the proportion of cells as a percentage of total CD45. (E) Heat map showing the median ArcSinh5-transformed marker expression values of the clusters identified in C and hierarchical clustering thereof. (F) Graph depicting the standard deviation in cell cluster frequencies between the technical replicate control samples (black circles) and the experimental decidual samples (red triangles). Noticeable is differential abundance of cluster 21 and 22 within CD4+ T cells, due to minor fluctuations in the expression of CD127, CD27, and CCR7.

FIGURE S4 Analysis of staining fluctuations between batches for the T cell CyTOF antibody panel. Ten replicate control samples from the same blood donor stained with the T cell CyTOF panel measured throughout the 7-month study period. **(A)** A t-SNE embedding showing the collective CD45+ cells (11.5 × 10^4^ cells) from 10 replicate control samples and 13 experimental decidual samples. Colored dots represent single cells from replicate samples and gray represents experimental samples. **(B)** Same t-SNE embedding as in panel A, colored for each replicate sample. **(C)** A t-SNE plot showing 20 cluster partitions in different colors. **(D)** Composition of the cell clusters in the individual samples (*n* = 23) represented in horizontal bars where the size of the colored segments represents the proportion of cells as a percentage of total CD45. **(E)** Heat map showing the median ArcSinh5-transformed marker expression values of the clusters identified in C and hierarchical clustering thereof. **(F)** Graph depicting the standard deviation in cell cluster frequencies between the technical replicate control samples (black circles) and the experimental decidual samples (red triangles). Noticeable is differential abundance of cluster 18 and 19 within CD4+ T cells, due to minor fluctuations in the expression of CD127, CD38, and CCR7.

FIGURE S5 Characterization of the innate lymphoid compartment. **(A)** Heatmap showing the marker expression values for the 14 identified NK and ILC clusters. Cluster IDs and cluster frequencies are displayed at the bottom of the heatmap. **(B)** Violin plots depicting expression values of indicated markers (arcSinh5-transformed) in the four tissue groups. **(C)** Heatmap showing the marker expression values for ILC clusters (CD3-CD7+) within the T cell panel. Only clusters expressing the co-inhibitory receptors CD39, TIM-3, TIGIT are depicted here. **(D)** Boxplots of sample frequencies, divided per trimester, of the clusters plotted as a fraction of total ILC. The Kruskal-Wallis with Dunn’s test for multiple comparisons was performed. **(E)** Cluster frequencies (as percentage of total ILC) of the depicted clusters. **(F)** PCA of the sample frequencies (as percentage of total ILC) where the gestational age groups are depicted along the first two components. The centroid of each group is indicated in gray. ^∗^*P* ≤ 0.05; ^∗∗^*P* ≤ 0.01; ^∗∗∗^*P* ≤ 0.005.

FIGURE S6 Characterization of TCRγδ T cells. **(A)** Heatmap showing the marker expression values for the seven identified TCRγδ cell clusters within the general panel (36 samples; 114,875 cells). Cluster IDs and cluster frequencies are displayed at the bottom of the heatmap. **(B)** First-level HSNE embedding of the expression values of the indicated markers. **(C)** Boxplots of sample frequencies, divided per trimester, of the clusters plotted as a fraction of total TCRγδ cells. The Kruskal-Wallis with Dunn’s test for multiple comparisons was performed. **(D)** PCA of the sample frequencies (as percentage of total TCRγδ cells) where the gestational age groups are depicted along the first two components. The centroid of each group is indicated in gray. ^∗^*P* ≤ 0.05; ^∗∗^*P* ≤ 0.01; ^∗∗∗^*P* ≤ 0.005.

FIGURE S7 In-depth characterization of the CD4+ Treg-like compartment where CD25+CD127- and CD25+CD127+ CD4+ T cells increase throughout gestation. **(A)** CD4+ NKT-like cell cluster identified within the general panel. **(B)** In-depth analysis of the regulatory-like CD4+ T cell (Treg-like) compartment, where the heatmap shows the marker expression values for the additional identified CD4+ Treg-like cell clusters within the T cell panel. Cluster IDs and cluster frequencies are displayed at the bottom of the heatmap. **(C)** Visualization of the five CD4+ Treg-like clusters, shown in Figure 3A, in a diffusion map along two components. Each color in the left panel represents a cluster of cells. In the right top panel, cells within the 1st trimester, 2nd trimester, and term decidua basalis and parietalis are portrayed. The bottom panel shows expression values of the specified markers in the diffusion map. **(D)** t-SNE embedding of the arcSinh-transformed expression values of CD127 and CD25 as observed in the three trimesters, measured by flow cytometry and gated within CD3+CD4+ T cells. CD4+CD25+CD127- clusters are circled in orange; CD4+CD25+CD127+ clusters are circled in blue.

FIGURE S8 Characterization of the CD8+ T cell compartment, including CD8+ NKT-like cells. **(A)** Heatmap showing the marker expression values of CD8+ NKT-like cell clusters identified within the general panel. **(B)** Fourth-level HSNE arcSinh5-transformed expression values of the specified markers, measured by flow cytometry and gated within CD3+CD8+ T cells. Colors in the left plot indicate tissue type (1st *n* = 3; 2nd *n* = 4; term basalis and parietalis *n* = 4). **(C)** Pie charts depicting the contribution of major subpopulations to the CD4+ and CD8+ T cell compartments.

FIGURE S9 Characterization of B cells. **(A)** Heatmap showing the marker expression values for the nine identified B cell clusters within the general panel (36 samples; 72,414 cells). **(B)** Percentage of CD20+ B cells in each trimester. **(C)** ArcSinh5-transformed expression values of the specified markers. ^∗^*P* ≤ 0.05; ^∗∗^*P* ≤ 0.01; ^∗∗∗^*P* ≤ 0.005.

TABLE S1 General CyTOF antibody panel^1^.

TABLE S2 T cell-specific CyTOF antibody panel^1^.

TABLE S3 Flow cytometry antibody panel^1^.
