# Supplementary figures and images for: Visualizing Dynamic Changes at the Maternal-Fetal Interface Throughout Human Pregnancy by Mass Cytometry
Source: Front Immunol. 2020 Oct 26;11:571300. doi: 10.3389/fimmu.2020.571300 (PMC7649376; doi:10.3389/fimmu.2020.571300)

**A**

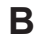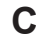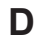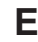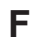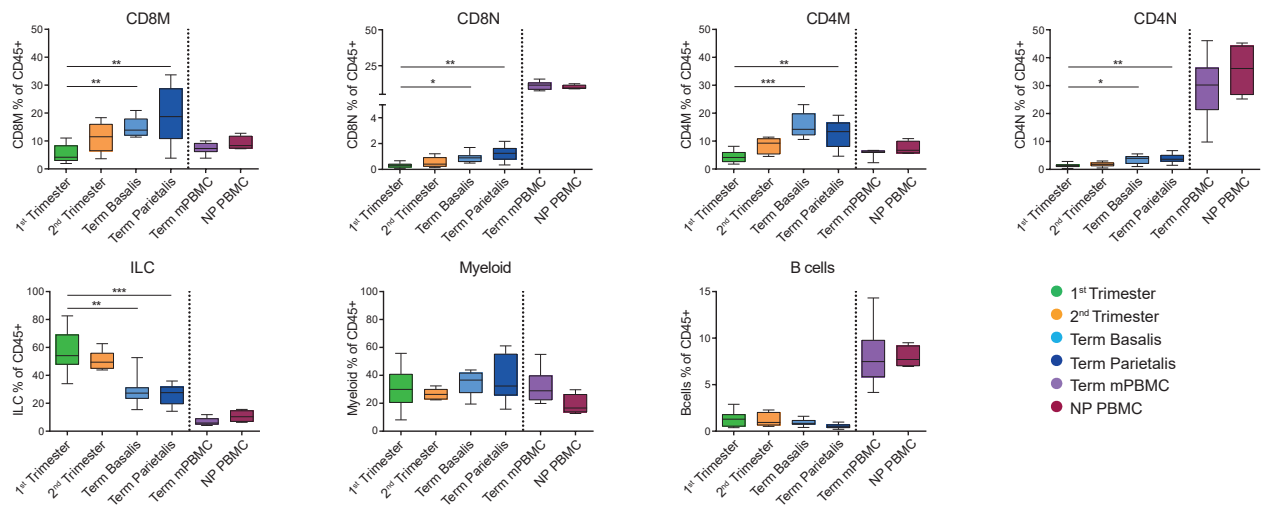

Supplement: FIGURE S1 — Identification of major immune cell lineages at the maternal-fetal interface using the T cell panel. (A) Gating strategy to select single, live CD45+ cells for downstream analysis. (B) Comparison of the absolute numbers and percentages of CD45+ cells measured by the general and the T cell panel. (C) Correlation plots of CD45+ cells measured by the general and T cell panel within the three trimesters, maternal peripheral blood mononuclear cells (mPBMC) and non-pregnant control samples (NP PBMC). (D) First-level HSNE visualization of the major immune cell lineages derived from decidua and peripheral blood. Colors top left indicate tissue type (1st trimester n = 11; 2nd trimester n = 5; term basalis and parietalis n = 8; mPBMC n = 8; NP PBMC n = 4); colors bottom left indicate major immune cell types (CD8M, CD8 memory T cells; CD8N, CD8 naïve T cells; CD4M, CD4 memory T cells; CD4N, CD4 naïve T cells); colors for plots on the right indicate the arcSinh5-transformed expression values of the specified markers where every dot represents a landmark. Memory and naïve clusters were distinguished based on CD45RO and CD45RA expression. (E) t-SNE visualization of the separation between decidual and peripheral blood samples (as percentage of CD45+ cells); every dot represents a single sample. (F) Major immune cell lineages (as percentage of CD45+ cells) throughout gestation and within mPBMC and NP PBMC. Boxplots depict the 10–90 percentile and the Kruskal-Wallis with Dunn’s test for multiple comparisons was applied. ∗P < 0.05; ∗∗P < 0.01; ∗∗∗P < 0.001. [file Image_1.pdf]

Figure S2

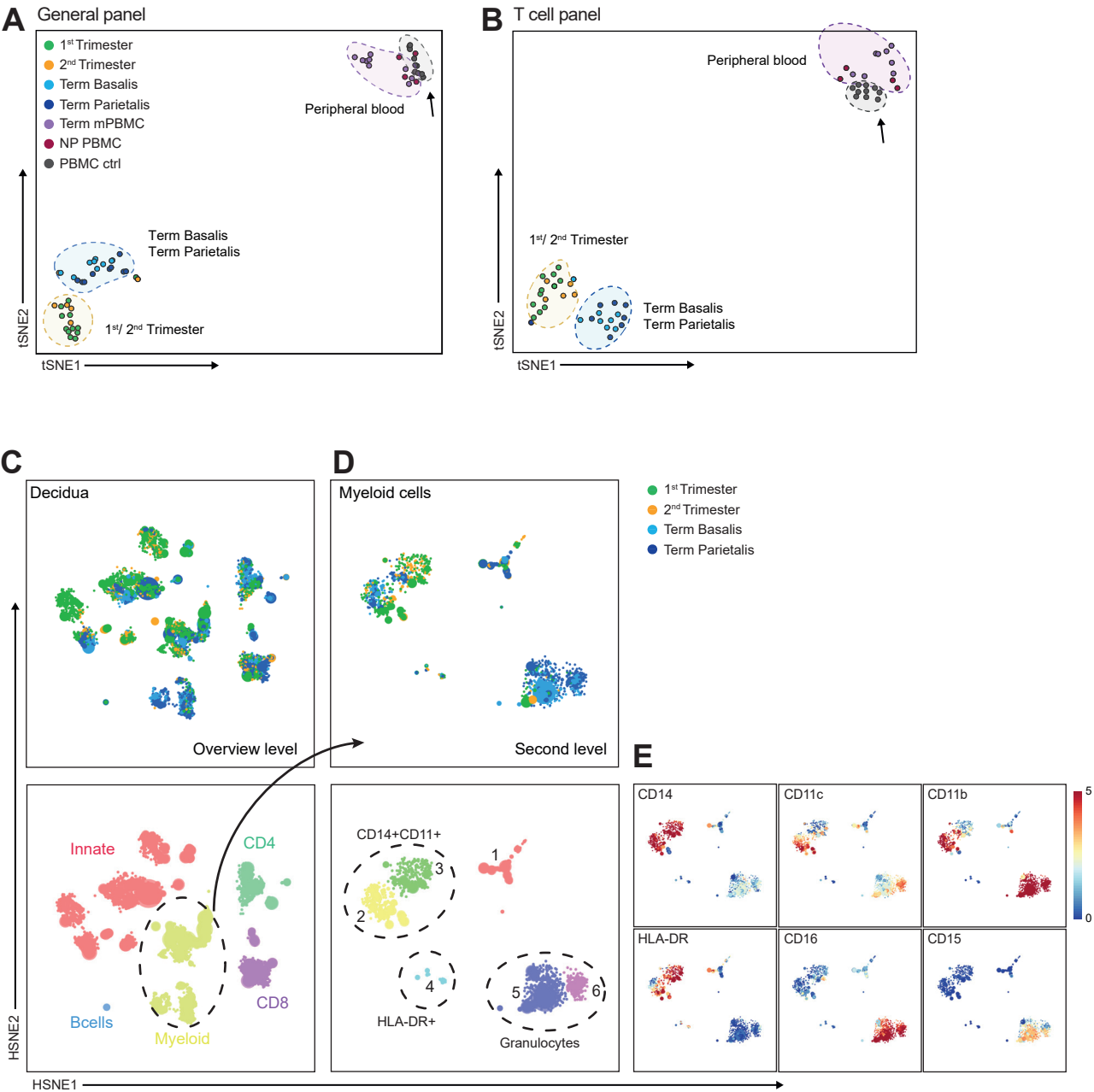

Supplement: FIGURE S2 — t-SNE visualization of PBMC reference samples and partitioning of the myeloid cell compartment into subpopulations. Cell frequencies (as percentage of CD45+ cells) are plotted where every dot represents a single sample within the general panel (A) and within the T cell panel (B). The gray arrow indicates the PBMC reference control samples clustering together. (C) HSNE overview (first) level embedding of all decidual samples with identification of the major immune cell lineages based on lineage marker expression. (D) Second-level HSNE embedding of the myeloid cells subdivided into six major subpopulations. (E) Second-level HSNE arcSinh5-transformed expression values of the specified markers where every dot represents a landmark. [file Image_2.pdf]

Figure S3

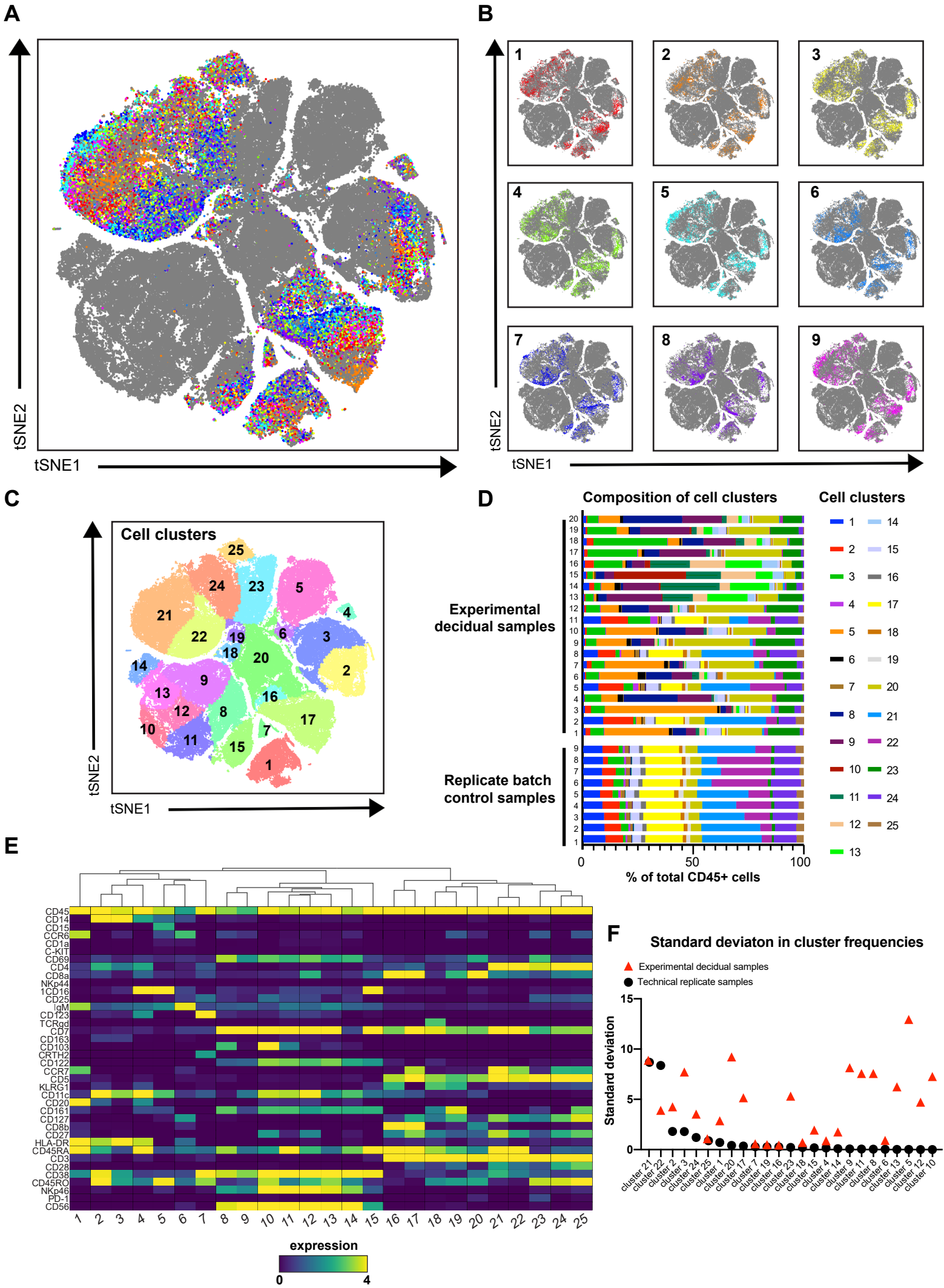

Supplement: FIGURE S3 — Analysis of staining fluctuations between batches for the general CyTOF antibody panel. Nine replicate control samples from the same blood donor stained with the general CyTOF panel measured throughout the 7-month study period. (A) A t-SNE embedding showing the collective CD45+ cells (14.5 × 104 cells) from nine replicate control samples and 20 experimental decidual samples. Colored dots represent single cells from replicate samples and gray represents experimental samples. (B) Same t-SNE embedding as in panel A, colored for each replicate sample. (C) A t-SNE plot showing 25 cluster partitions in different colors. (D) Composition of the cell clusters in the individual samples (n = 29) represented in horizontal bars where the size of the colored segments represents the proportion of cells as a percentage of total CD45. (E) Heat map showing the median ArcSinh5-transformed marker expression values of the clusters identified in C and hierarchical clustering thereof. (F) Graph depicting the standard deviation in cell cluster frequencies between the technical replicate control samples (black circles) and the experimental decidual samples (red triangles). Noticeable is differential abundance of cluster 21 and 22 within CD4+ T cells, due to minor fluctuations in the expression of CD127, CD27, and CCR7. [file Image_3.pdf]

Figure S4

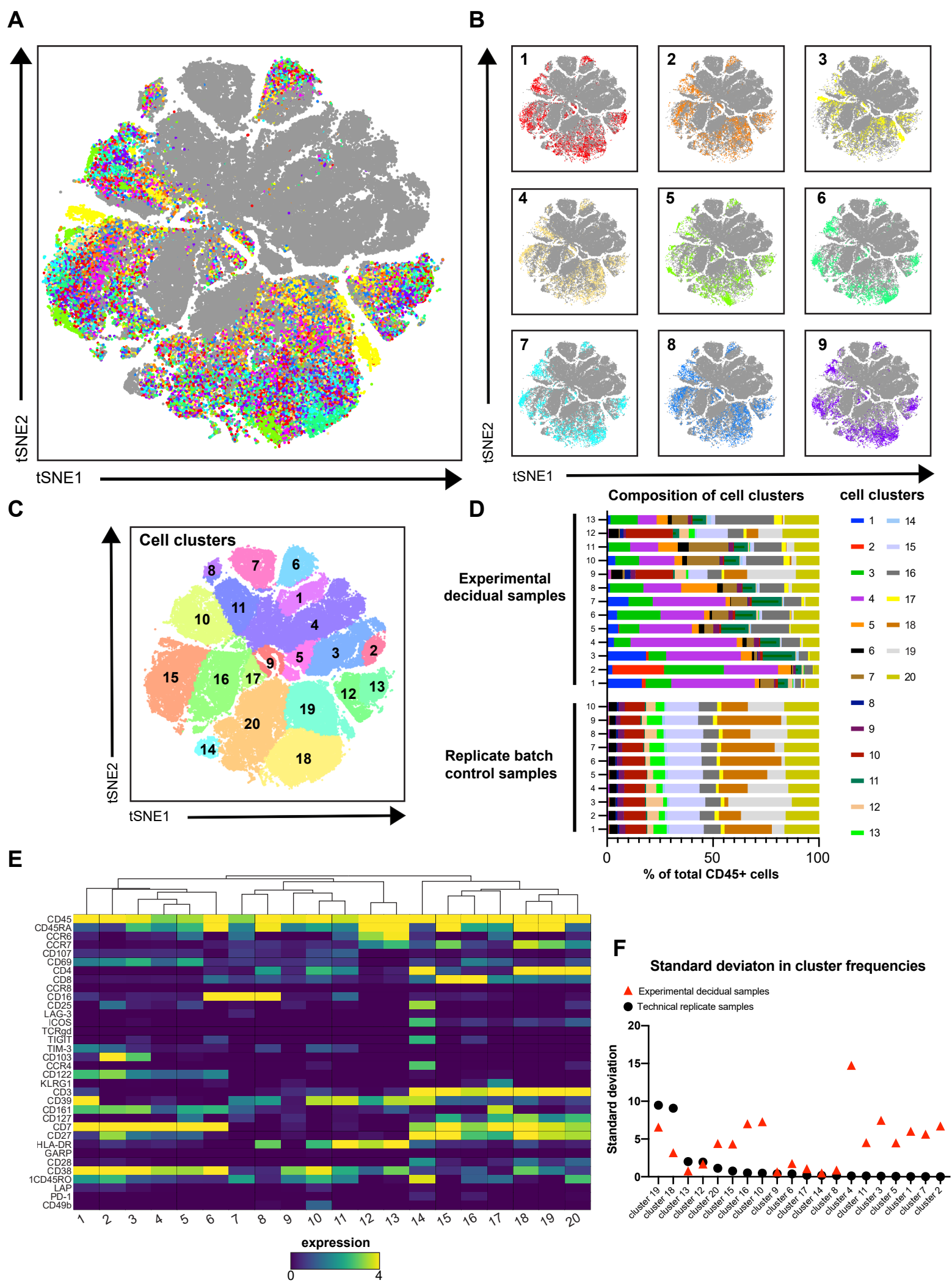

Supplement: FIGURE S4 — Analysis of staining fluctuations between batches for the T cell CyTOF antibody panel. Ten replicate control samples from the same blood donor stained with the T cell CyTOF panel measured throughout the 7-month study period. (A) A t-SNE embedding showing the collective CD45+ cells (11.5 × 104 cells) from 10 replicate control samples and 13 experimental decidual samples. Colored dots represent single cells from replicate samples and gray represents experimental samples. (B) Same t-SNE embedding as in panel A, colored for each replicate sample. (C) A t-SNE plot showing 20 cluster partitions in different colors. (D) Composition of the cell clusters in the individual samples (n = 23) represented in horizontal bars where the size of the colored segments represents the proportion of cells as a percentage of total CD45. (E) Heat map showing the median ArcSinh5-transformed marker expression values of the clusters identified in C and hierarchical clustering thereof. (F) Graph depicting the standard deviation in cell cluster frequencies between the technical replicate control samples (black circles) and the experimental decidual samples (red triangles). Noticeable is differential abundance of cluster 18 and 19 within CD4+ T cells, due to minor fluctuations in the expression of CD127, CD38, and CCR7. [file Image_4.pdf]

Figure S5

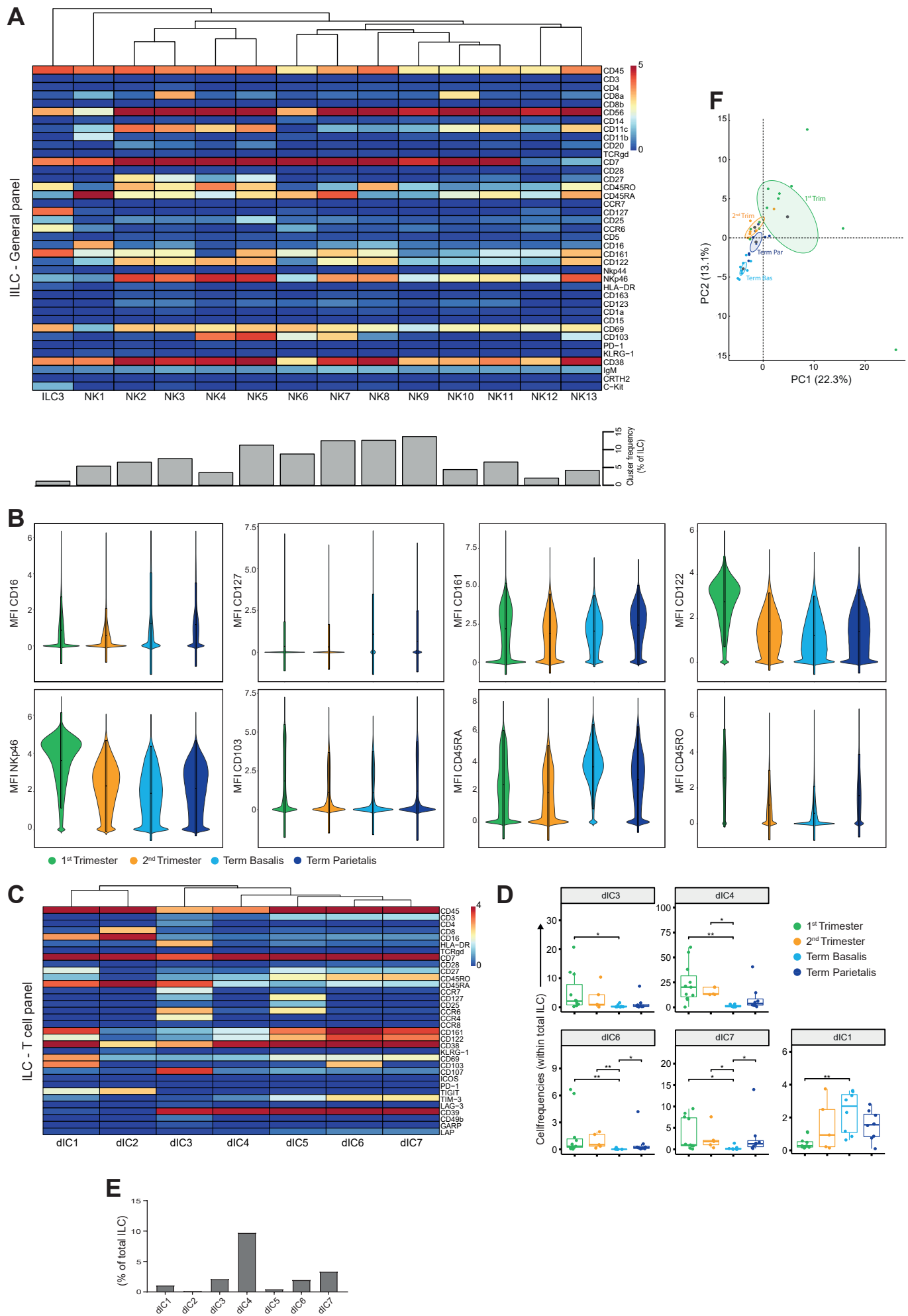

Supplement: FIGURE S5 — Characterization of the innate lymphoid compartment. (A) Heatmap showing the marker expression values for the 14 identified NK and ILC clusters. Cluster IDs and cluster frequencies are displayed at the bottom of the heatmap. (B) Violin plots depicting expression values of indicated markers (arcSinh5-transformed) in the four tissue groups. (C) Heatmap showing the marker expression values for ILC clusters (CD3-CD7+) within the T cell panel. Only clusters expressing the co-inhibitory receptors CD39, TIM-3, TIGIT are depicted here. (D) Boxplots of sample frequencies, divided per trimester, of the clusters plotted as a fraction of total ILC. The Kruskal-Wallis with Dunn’s test for multiple comparisons was performed. (E) Cluster frequencies (as percentage of total ILC) of the depicted clusters. (F) PCA of the sample frequencies (as percentage of total ILC) where the gestational age groups are depicted along the first two components. The centroid of each group is indicated in gray. ∗P ≤ 0.05; ∗∗P ≤ 0.01; ∗∗∗P ≤ 0.005. [file Image_5.pdf]

# Figure S6

**A**

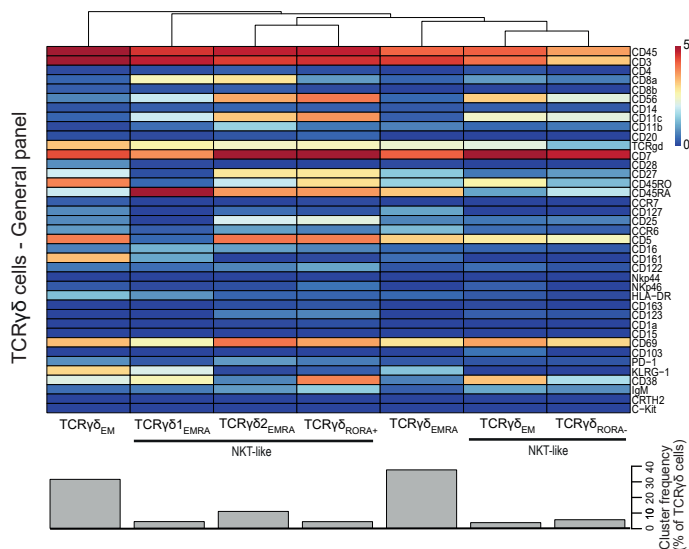

**B**

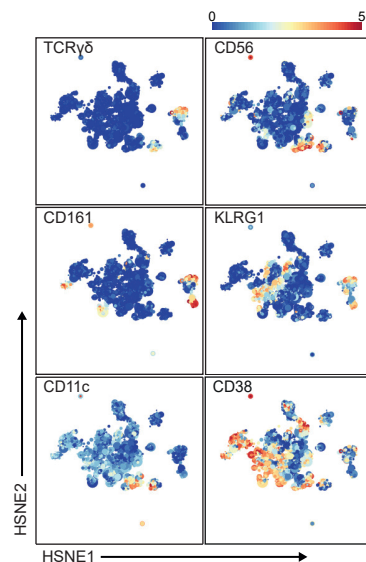

**C**

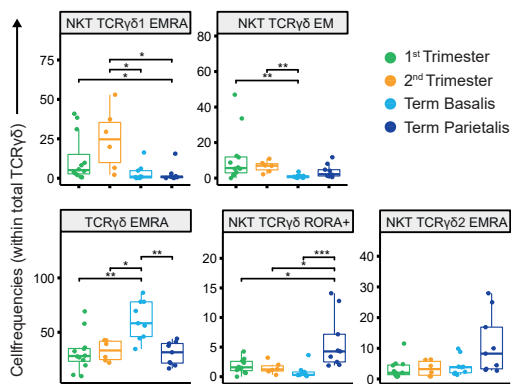

**D**

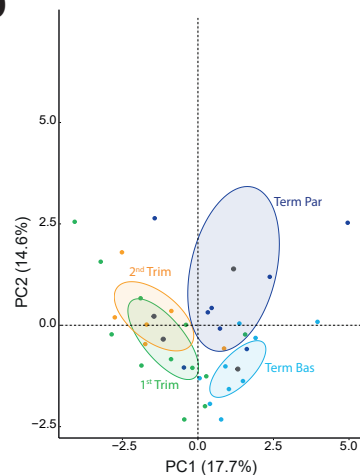

Supplement: FIGURE S6 — Characterization of TCRγδ T cells. (A) Heatmap showing the marker expression values for the seven identified TCRγδ cell clusters within the general panel (36 samples; 114,875 cells). Cluster IDs and cluster frequencies are displayed at the bottom of the heatmap. (B) First-level HSNE embedding of the expression values of the indicated markers. (C) Boxplots of sample frequencies, divided per trimester, of the clusters plotted as a fraction of total TCRγδ cells. The Kruskal-Wallis with Dunn’s test for multiple comparisons was performed. (D) PCA of the sample frequencies (as percentage of total TCRγδ cells) where the gestational age groups are depicted along the first two components. The centroid of each group is indicated in gray. ∗P ≤ 0.05; ∗∗P ≤ 0.01; ∗∗∗P ≤ 0.005. [file Image_6.pdf]

**A**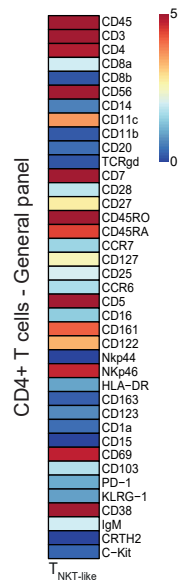**B**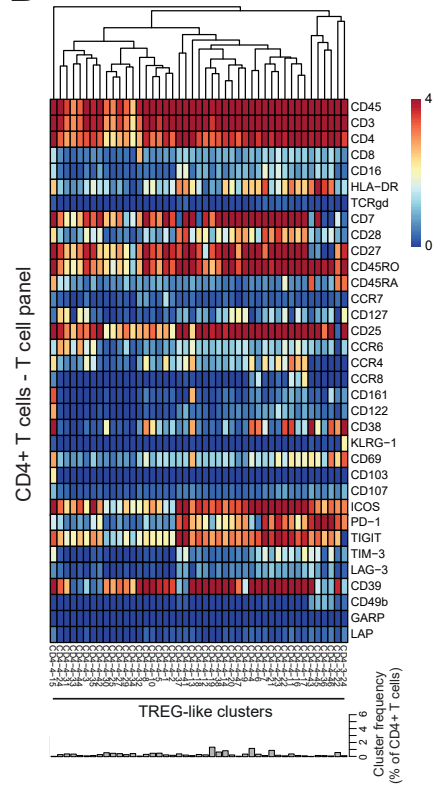**C**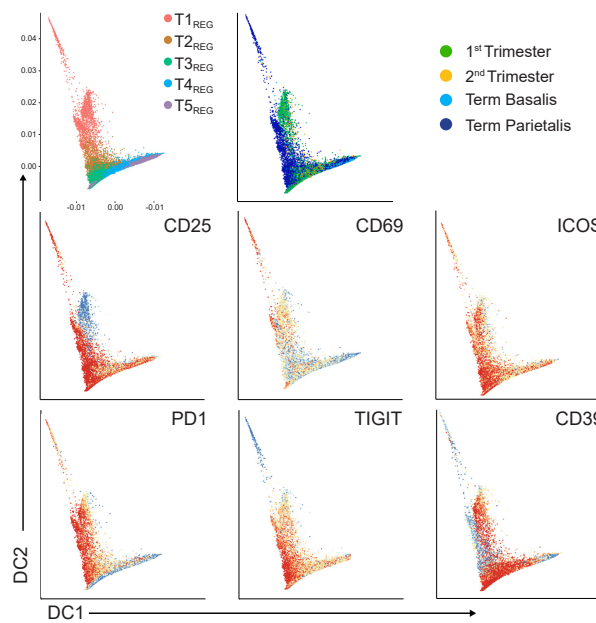**D**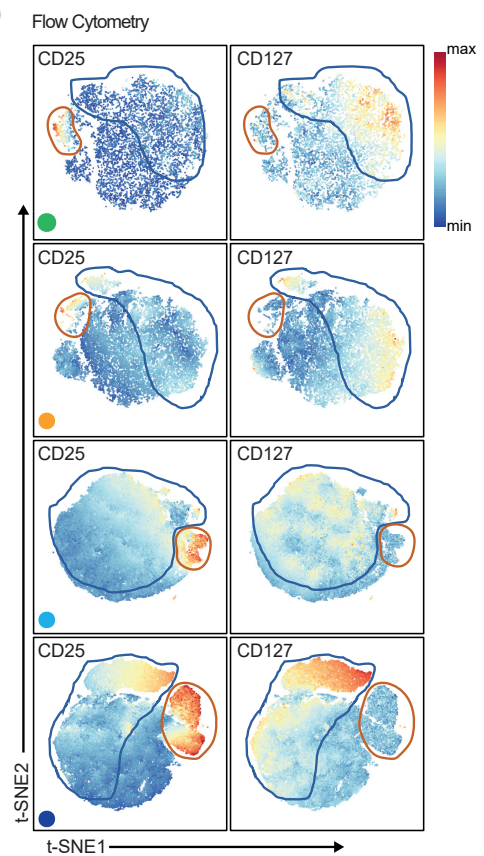

Supplement: FIGURE S7 — In-depth characterization of the CD4+ Treg-like compartment where CD25+CD127- and CD25+CD127+ CD4+ T cells increase throughout gestation. (A) CD4+ NKT-like cell cluster identified within the general panel. (B) In-depth analysis of the regulatory-like CD4+ T cell (Treg-like) compartment, where the heatmap shows the marker expression values for the additional identified CD4+ Treg-like cell clusters within the T cell panel. Cluster IDs and cluster frequencies are displayed at the bottom of the heatmap. (C) Visualization of the five CD4+ Treg-like clusters, shown in Figure 3A, in a diffusion map along two components. Each color in the left panel represents a cluster of cells. In the right top panel, cells within the 1st trimester, 2nd trimester, and term decidua basalis and parietalis are portrayed. The bottom panel shows expression values of the specified markers in the diffusion map. (D) t-SNE embedding of the arcSinh-transformed expression values of CD127 and CD25 as observed in the three trimesters, measured by flow cytometry and gated within CD3+CD4+ T cells. CD4+CD25+CD127- clusters are circled in orange; CD4+CD25+CD127+ clusters are circled in blue. [file Image_7.pdf]

# Figure S8

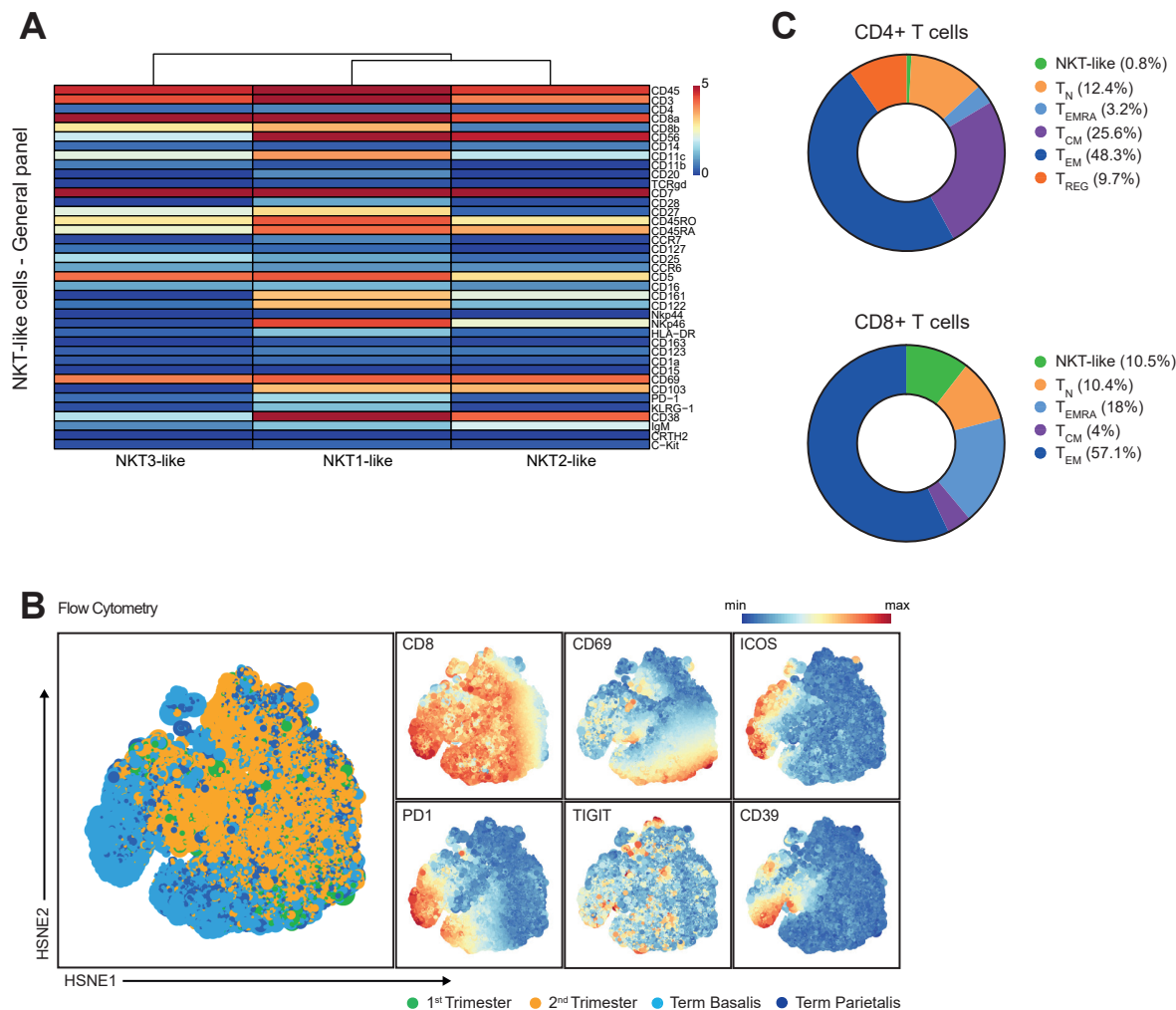

Supplement: FIGURE S8 — Characterization of the CD8+ T cell compartment, including CD8+ NKT-like cells. (A) Heatmap showing the marker expression values of CD8+ NKT-like cell clusters identified within the general panel. (B) Fourth-level HSNE arcSinh5-transformed expression values of the specified markers, measured by flow cytometry and gated within CD3+CD8+ T cells. Colors in the left plot indicate tissue type (1st n = 3; 2nd n = 4; term basalis and parietalis n = 4). (C) Pie charts depicting the contribution of major subpopulations to the CD4+ and CD8+ T cell compartments. [file Image_8.pdf]

# Figure S9

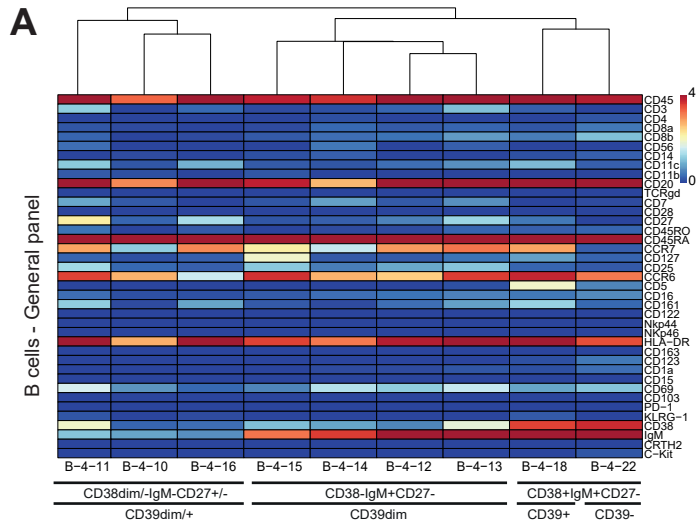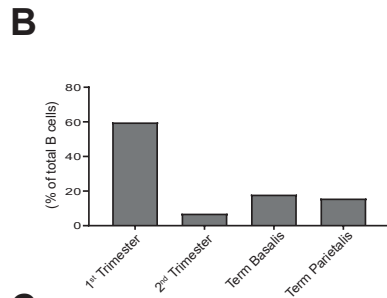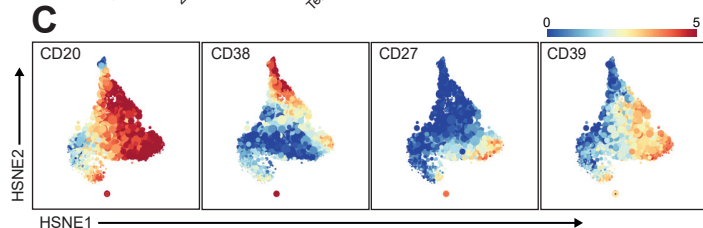

Supplement: FIGURE S9 — Characterization of B cells. (A) Heatmap showing the marker expression values for the nine identified B cell clusters within the general panel (36 samples; 72,414 cells). (B) Percentage of CD20+ B cells in each trimester. (C) ArcSinh5-transformed expression values of the specified markers. ∗P ≤ 0.05; ∗∗P ≤ 0.01; ∗∗∗P ≤ 0.005. [file Image_9.pdf]
