# Supplementary material for: Visualizing Dynamic Changes at the Maternal-Fetal Interface Throughout Human Pregnancy by Mass Cytometry
Source: Front Immunol. 2020 Oct 26;11:571300. doi: 10.3389/fimmu.2020.571300 (PMC7649376; doi:10.3389/fimmu.2020.571300)
Supplement: TABLE S1 — General CyTOF antibody panel1. [file Table_1.docx]

**Supplementary Table 1. General CyTOF antibody panel ^1^**

Antigen Tag Clone Company Cat#

1 CD8a 146Nd RPA-T8 Fluidigm 3146001B

2 CD11c 162Dy Bu15 Fluidigm 3162005B

3 CD127 165Ho AO19D5 Fluidigm 3165008B

4 CD38 172Yb HIT2 Fluidigm 3172007B

5 CD69 144Nd FN50 Fluidigm 3144018B

6 CD11b 209Bi ICRF44 Fluidigm 3209003B

7 CD45 89Y HI30 Fluidigm 3089003B

8 CCR6 141Pr G034E3 Fluidigm 3141003A

9 C-Kit 143Nd 104D2 Fluidigm 3143001B

10 CD4 145Nd RPA-T4 Fluidigm 3145001B

11 CD16 148Nd 3G8 Fluidigm 3148004B

12 CD25 149Sm 2A3 Fluidigm 3149010B

13 CD123 151Eu 6H6 Fluidigm 3151001B

14 CD7 153Eu CD7-6B7 Fluidigm 3153014B

15 CD163 154Sm GHI/61 Fluidigm 3154007B

16 CCR7 159Tb G043H7 Fluidigm 3159003A

17 CD161 164Dy HP-3G10 Fluidigm 3164009B

18 CD27 167Er O323 Fluidigm 3167002B

19 CD45RA 169Tm HI100 Fluidigm 3169008B

20 CD3 170Er UCHT1 Fluidigm 3170001B

21 PD-1 175Lu EH 12.2H7 Fluidigm 3175008B

22 CD56 176Yb NCAM16.2 Fluidigm 3176008B

23 TCRγδ 152Sm 11F2 Fluidigm 3152008B

24 CD15 115In W6D3 Biolegend 323035

25 CD1a 142Nd HI149 Sony 2100510

26 CD5 160Gd UCHT2 Biolegend 300627

27 HLA-DR 168Er L243 Biolegend 307651

28 IgM 150Nd MHM88 Biolegend 314527

29 CD103 155Gd Ber-ACT8 Biolegend 350202

30 CRTH2 156Gd BM16 Biolegend 350102

31 CD20 163Dy 2H7 Biolegend 302343

32 CD28 171Yb CD28.2 Biolegend 302937

33 CD45RO 173Yb UCHL1 Biolegend 304239

34 CD122 158Gd TU27 Biolegend 339015

35 KLRG-1 161Dy REA261 MACS 120-014-229

36 CD8b 166Er SIDI8BEE Ebioscience 14-5273

37 NKp46 174Yb 9E 2 Biolegend 331902

38 Nkp44 147Sm 253415 R&D Systems MAB22491

39 CD14 Qdot800 TüK4 ThermoFisher/ Q10064

Invitrogen

^1^ 36 antibodies published in Van Unen et al. Immunity 2016 and Li et al. J Exp Med 2018, with addition of CD69, CD5, CD15, and CD1a.

**Supplementary Table 2. T cell-specific CyTOF antibody panel ^1^**

Antigen Tag Clone Company Cat#

1 CD3 161Dy UCHT1 Biolegend 300443

2 CD4 145Nd RPA-T4 Fluidigm 3145001B

3 CD7 166Er M-T701 Fluidigm 3166027B

4 CD8a 146Nd RPA-T8 Fluidigm 3146001B

5 CD16 148Nd 3G8 Fluidigm 3148004B

6 CD20 163Dy 2H7 Biolegend 302343

7 CD25 149Sm 2A3 Fluidigm 3149010B

8 CD27 167Er O323 Fluidigm 3167002B

9 CD28 171Yb CD28.2 Biolegend 302902

10 CD38 172Yb HIT2 Fluidigm 3172007B

11 CD39 162Dy A1 Biolegend 328202

12 CD45 89Y HI30 Fluidigm 3089003B

13 GARP 169Tm 7B11 Biolegend 353502

14 CD45RO 173Yb UCHL1 Biolegend 304239

15 CD49b 176Yb P1e6c5 Biolegend 359301

16 CD69 144Nd FN50 Fluidigm 3144018B

17 CD103 155Gd Ber-ACT8 Biolegend 350202

18 CD107 143Nd H4A3 Biolegend 14-1079

19 CD122 158Gd TU27 Biolegend 339002

20 CD127 165Ho AO19D5 Fluidigm 3165008B

21 CTLA-4 ^2^ 170Er 14D3 Fluidigm 3170005B

22 CD161 164Dy HP-3G10 Fluidigm 3164009B

23 CCR4 156Gd L291H4 Biolegend 359402

24 CCR6 141Pr G034E3 Fluidigm 3141003A

25 CCR7 142Nd G043H7 Biolegend 353237

26 PD-1 175Lu EH 12.2H7 Fluidigm 3175008B

27 LAG3 150Nd 874501 Fluidigm 3150016B

28 ICOS 151Eu C398.4A Fluidigm 3151020B

29 CCR8 147Sm L263G8 Biolegend 360602

30 LAP 174Yb TW4-2F8 Biolegend 349602

31 GITR ^2^ 159Tb 621 Fluidigm 3159020B

32 TIM-3 154Sm F38-2E2 Fluidigm 3154010B

33 TIGIT 153Eu MBSA43 Fluidigm 3153019B

34 HLA-DR 168Er L243 Biolegend 307651

35 TCRγδ 152Sm 11F2 Fluidigm 3152008B

36 KLRG1 160Gd REA261 Miltenyi 120-014-229

37 CD45RA Qdot655 MEM-56 ThermoFisher/ Q10069

Invitrogen

^1^ Part of mass cytometry panel published in Laban et al. Plos One 2018.

^2^ GITR and CTLA-4 antibodies were validated, but these markers did not show expression in decidual samples.

**Supplementary Table 3. Flow cytometry antibody panel ^1^**

Antigen Fluorochrome Clone Company Cat#

Extracellular

1 CD45 Krome Orange J.33 Beckman Coulter B36294

2 CD3 ECD UCHT1 Beckman Coulter A07748

3 CD3 PE SK7 BD Biosciences 345765

4 CD4 A700 RPA-T4 BD Biosciences 557922

5 CD8 Pacific Blue RPA-T8 BD Biosciences 558207

6 CD127 PerCP-Cy5.5 A019D5 Biolegend 351321

7 CD25 PE 2A3 BD Biosciences 341011

8 ICOS APC-Cy7 C398.4A Biolegend 313529

9 TIGIT APC MBSA43 Invitrogen 17-9500-41

10 CD39 BV510 A1 Biolegend 328219

11 PD-1 PE-Cy7 EH12.1 BD Biosciences 561272

12 CD69 PE FN50 BD Biosciences 555531

13 CCR7 A488 G043H7 Biolegend 353206

14 CD45RA PE-TexasRed MEM-56 Life Technologies MHCD45RA17

Intracellular

15 FOXP3 FITC PCH101 Invitrogen 11-4776-71

16 HELIOS Pacific Blue 22F6 Biolegend 137220

17 CTLA-4 PE-Cy7 L3D10 Biolegend 349913

^1^ Matched IgG controls were included.
